# Correction to: European headache federation guideline on the use of monoclonal antibodies acting on the calcitonin gene related peptide or its receptor for migraine prevention

**DOI:** 10.1186/s10194-019-0972-5

**Published:** 2019-05-23

**Authors:** Simona Sacco, Lars Bendtsen, Messoud Ashina, Uwe Reuter, Gisela Terwindt, Dimos-Dimitrios Mitsikostas, Paolo Martelletti

**Affiliations:** 1 Neuroscience Section, Department of Applied Clinical Sciences and Biotechnology, University of L’Aquila, via Vetoio, 67100 L’Aquila, Italy; 2 Department of Neurology, Danish Headache Center, Rigshospitalet Glostrup, Faculty of Health and Medical Sciences, University of Copenhagen, Copenhagen, Denmark; 3 Department of Neurology, Charité Universitätsmedizin Berlin, Berlin, Germany; 4 Department of Neurology, Leiden University Medical Center, Leiden, The Netherlands; 5 1st Department of Neurology, National and Kapodistrian University of Athens, Athens, Greece; 6 Department of Clinical and Molecular Medicine, Sapienza University, Rome, Italy

**Correction to: J Headache Pain (2019) 20:6**

**https://doi.org/10.1186/s10194-018-0955-y**

Following publication of the original article [1], the authors notified us of some misreported data due to the publication of the EVOLVE-2 trial (Cephalalgia. 2018;38:1442–1454), which substantially changed the level of evidence of galcanezumab for the prevention of episodic migraine. All changes are marked in bold throughout the text, as well as in the figure captions. Please note that the final recommendations remain unchanged.

Reference #51 was added: Skljarevski V, Matharu M, Millen BA, Ossipov MH, Kim B-K, Yang JY. Efficacy and safety of galcanezumab for the prevention of episodic migraine: Results of the EVOLVE-2 Phase 3 randomized controlled clinical trial. Cephalalgia. 2018;38:1442-1454.

Please find below the updated text, tables, and figures.

## Results

We identified **29** studies eligible to be considered in the present guidelines (Fig. 1) [23–**51**]. **Fifteen** of the selected studies (Tables 1 and 2) were phase II or III randomized clinical trials (RCTs) reporting data on safety or efficacy of the CGRP mAbs [26,27,31-36,41-45,50,**51**]; 14 additional studies were post-hoc or pooled analyses from the RCTs, open label-extension of the RCTs, or open label studies [23–25,28-30,37-40,46–49]. Risk of bias summary for the selected studies is reported in Fig. 2. Certainty assessment of outcomes for studies in EM and CM is reported in Tables 3 and 4. Recommendations related to the use of CGRP mAbs for prevention of EM and CM are reported in Table 5.

**Fig. 1:**
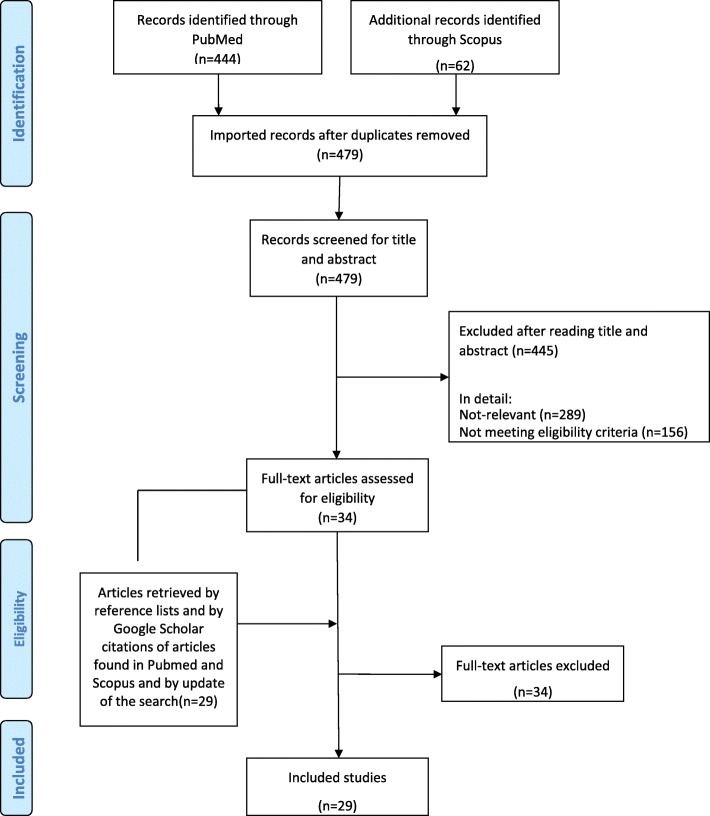
Process of identifying eligible studies for the guideline. The number of included studies has been modified from 28 to 29

**Table 1:** Characteristics of the randomized placebo-controlled trials considered for the guideline in episodic migraine

Study	Study phase	Treatment regimen	Duration of treatment	Participants (n)	Women (%)	Age range (years)	Monthly migraine days (range)	Preventive treatment (% using)	Exclusion by preventive failure (n. of drugs/ categories)
Eptinezumab									
Dodick, 2014 [32]	II	1000 mg quarterly ev	3 months	174	80–83	18–55	5–14	Not Allowed	–
Erenumab									
Sun, 2016 [44]	II	70 mg monthly sc	3 months	483	77–83	18–60	4–14	Not allowed	> 2
STRIVE [36]	III	70 mg monthly sc 140 mg monthly sc	6 months	955	85–86	18–65	4–14	2–3	> 2
ARISE [35]	III	70 mg monthly sc	3 months	577	85–86	18–65	4–14	6–7	> 2
Fremanezumab									
Bigal, 2015 [27]	II	225 mg (225 mg quarterly) sc 675 mg (225 mg monthly) sc	3 months	297	85–91	18–65	8–14	27–34	> 2
HALO EM [34]	III	225 mg monthly sc 675 mg quarterly sc	3 months	875	84–86	18–70	6–14	20–21	≥2
Galcanezumab									
Dodick, 2014 [33]	II	150 mg every two weeks sc	3 months	218	82–87	18–65	4–14	Not Allowed	> 2
Skljarevski, 2018 [42]	II	120 mg monthly sc 300 mg monthly sc	3 months	410	80–85	18–65	4–14	NR	> 2
EVOLVE-1 [43]	III	120 mg monthly sc (240 mg ld) 240 mg monthly sc	6 months	858	83–85	18–65	4–14	Not allowed	> 2
EVOLVE-2 [51]	III	120 mg monthly sc (240 mg ld) 240 mg monthly sc	6 months	922	85–86	18–65	4–14	Not allowed	> 2

ev: endovenous; sc: subcutaneous; ld loading dose; NR: not reported.

Reported treatment regimen in the Fremanezumab study is 225 mg monthly.

For the Galcanezumab study, the originally published line for EVOLVE-2 was replaced with Skljarevski, 2018. A new row was introduced for EVOLVE-2, linked to reference 51

**Table 2:** Characteristics of the randomized placebo-controlled trials considered for the guideline in chronic migraine

Study	Study phase	Treatment regimen	Duration of treatment	Participants (n)	Women (%)	Age range (years)	Definition of chronic migraine	Preventive treatment (% using)	Exclusion by preventive failure (n. of drugs/ categories)
Erenumab									
Tepper, 2017 [45]	II	70 mg monthly sc 140 mg monthly sc	3 months	667	79–87	18–65	ICHD-3, beta version	Not allowed	> 3
Fremanezumab									
Bigal, 2015 [26]	II	225 mg monthly sc (675 mg ld)	3 months	264	85–86	18–65	ICHD-3, beta version	38–43	> 3
HALO CM [41]	III	225 mg monthly sc (675 mg ld) 675 mg quarterly sc	3 months	1130	87–88	18–70	ICHD-3, beta version	20–22	≥2
Galcanezumab									
REGAIN [31]	III	120 mg monthly sc (240 mg ld) 240 mg monthly sc	3 months	1117	82–87	18–65	ICHD-3, beta version (required at least 1 headache-free day per month)	13–16	> 2

sc: subcutaneous; ld loading dose

**Fig. 2:**
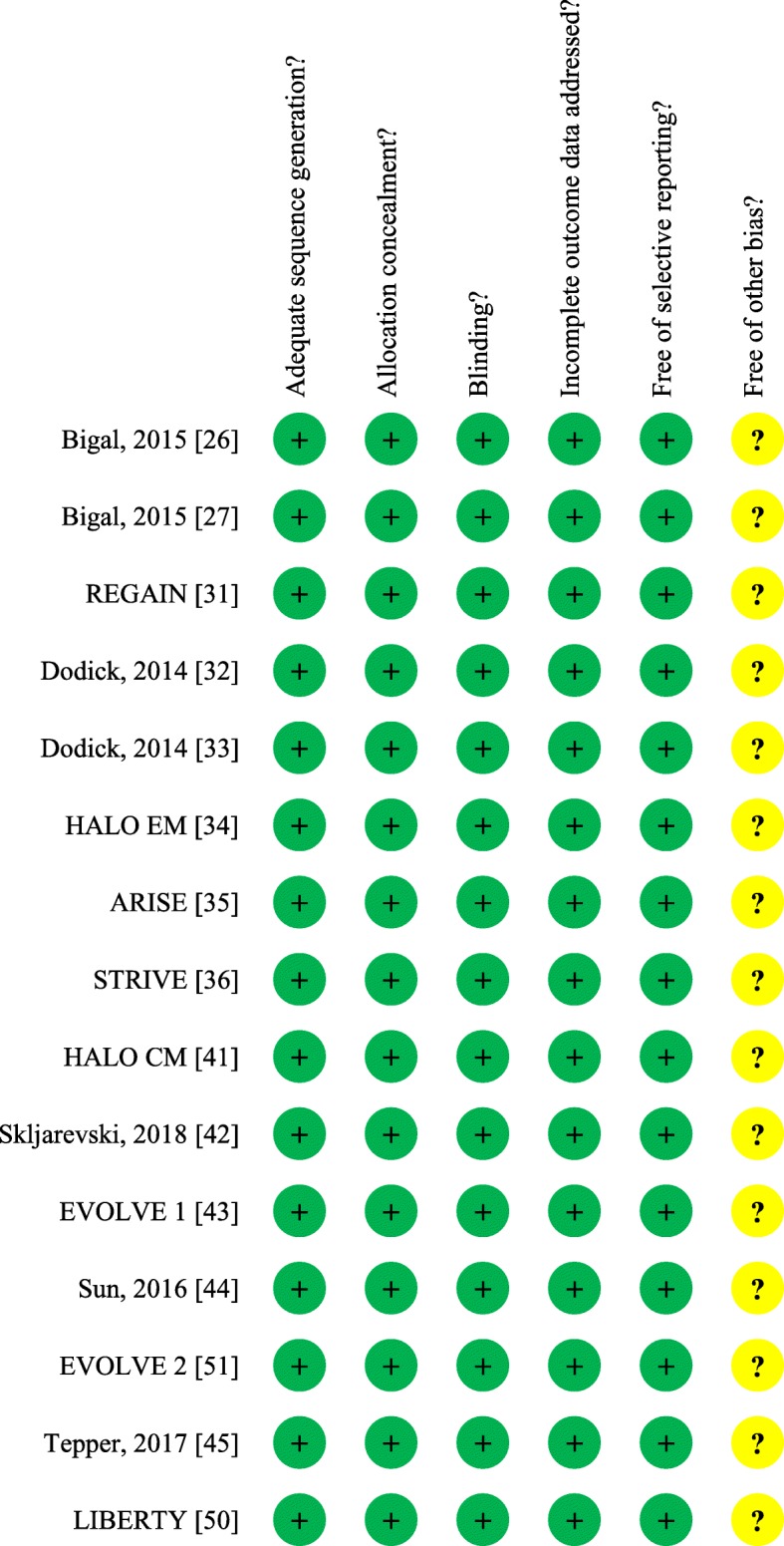
Risk of bias summary for the studies considered for the guideline. The EVOLVE 2 line was replaced wi Skljarevski, 2018. A new line was introduced for EVOLVE 2 (linked to reference 51) right under Sun, 2016

**Table 3:** Certainty in the assessment of efficacy outcomes for anti-calcitonin gene-related peptide monoclonal antibodies for prevention in episodic migraine

	Certainty assessment							Certainty
	Number of studies	Study design	Risk of bias	Inconsistency	Indirectness	Imprecision	Other considerations	
Eptinezumab								
1000 mg quarterly ev	1	RCT	not serious	serious^a^	not serious	serious^b^	none	⨁⨁◯◯ LOW
Erenumab								
70 monthly sc (except functional improvement)	3	RCT	not serious	not serious	not serious	not serious	none	⨁⨁⨁⨁ HIGH
70 monthly sc (functional improvement)	1	RCT	not serious	serious^a^	not serious	not serious	none	⨁⨁⨁◯ MEDIUM
140 monthly sc	1	RCT	not serious	serious^a^	not serious	not serious	none	⨁⨁⨁◯ MEDIUM
Fremanezumab								
225 monthly sc	2	RCT	not serious	not serious	not serious	not serious	none	⨁⨁⨁⨁ HIGH
675 quarterly sc	1	RCT	not serious	serious^a^	not serious	not serious	none	⨁⨁⨁◯ MEDIUM
Galcanezumab								
240 mg ld + 120 mg monthly sc	2	RCT	not serious	not serious	not serious	not serious	none	⨁⨁⨁⨁ HIGH
240 mg monthly sc	2	RCT	not serious	not serious	not serious	not serious	none	⨁⨁⨁⨁ HIGH

sc: subcutaneous; ev: endovenous; RCT: randomized controlled trial. ^a^Inconsistency because of lack of replication; ^b^Imprecision because of exploratory study. The inconsistency for the Galcanezumab study was changed from serios to not serios, and the certainty from medium to high

**Table 4:** Certainty in the assessment of efficacy outcomes for anti-calcitonin gene-related peptide monoclonal antibodies for prevention in chronic migraine

	Certainty assessment							Certainty
	Number of studies	Study design	Risk of bias	Inconsistency	Indirectness	Imprecision	Other considerations	
Erenumab								
70 monthly sc	1	RCT	not serious	serious^a^	not serious	not serious	none	⨁⨁⨁◯ MEDIUM
140 monthly sc	1	RCT	not serious	serious^a^	not serious	not serious	none	⨁⨁⨁◯ MEDIUM
Fremanezumab								
675 quarterly sc	1	RCT	not serious	serious^a^	not serious	not serious	none	⨁⨁⨁◯ MEDIUM
675 ld + 225 quarterly sc (except functional improvement)	2	RCT	not serious	not serious	not serious	not serious	none	⨁⨁⨁⨁ HIGH
675 ld + 225 quarterly sc (functional improvement)	1	RCT	not serious	serious^a^	not serious	not serious	none	⨁⨁⨁◯ MEDIUM
Galcanezumab								
240 mg ld + 120 mg monthly sc	1	RCT	not serious	serious^a^	not serious	not serious	none	⨁⨁⨁◯ MEDIUM
240 mg monthly sc	1	RCT	not serious	serious^a^	not serious	not serious	none	⨁⨁⨁◯ MEDIUM

^a^Inconsistency because of lack of replication

*sc* subcutaneous, *ld* loading dose, *RCT* randomized controlled trial

**Table 5:** Recommendations on the use of calcitonin gene-related peptide monoclonal antibodies for the prevention of episodic and chronic migraine

Setting	Drug	Recommendation	Quality of evidence	Strength of the recommendation
Migraine prevention in patients with episodic migraine	Eptinezumab 1000 mg quarterly	Suggested	⨁⨁◯◯ LOW	↑? Weak
	Erenumab 70 mg monthly	Recommended	⨁⨁⨁⨁ HIGH	↑↑ Strong
	Erenumab 140 mg monthly	Recommended	⨁⨁⨁◯ MEDIUM	↑↑Strong
	Fremanezumab 225 mg monthly	Recommended	⨁⨁⨁⨁ HIGH	↑↑ Strong
	Fremanezumab 675 mg quarterly	Recommended	⨁⨁⨁◯ MEDIUM	↑↑Strong
	Galcanezumab 240 mg loading dose + 120 mg monthly	Recommended	⨁⨁⨁⨁ HIGH	↑↑ Strong
	Galcanezumab 240 mg monthly	Recommended	⨁⨁⨁⨁ HIGH	↑↑ Strong
Migraine prevention in patients with chronic migraine	Erenumab 70 mg monthly	Recommended	⨁⨁⨁◯ MEDIUM	↑↑Strong
	Erenumab 140 mg monthly	Recommended	⨁⨁⨁◯ MEDIUM	↑↑Strong
	Fremanezumab 675 mg quarterly	Recommended	⨁⨁⨁◯ MEDIUM	↑↑Strong
	Fremanezumab 675 mg loading dose + 225 mg monthly	Recommended	⨁⨁⨁⨁ HIGH	↑↑ Strong
	Galcanezumab 240 mg loading dose + 120 mg monthly	Recommended	⨁⨁⨁◯ MEDIUM	↑↑Strong
	Galcanezumab 240 mg monthly	Recommended	⨁⨁⨁◯ MEDIUM	↑↑Strong

Symbols depict the strength of the recommendation according to the GRADE system. The quality of evidence for the Galcanezumab study was changed from medium to high

**PICO question 1:** In patients with EM, is preventive treatment with CGRP mAbs as compared to placebo, effective and safe?

*Population*: patients with EM

*Intervention*: any preventive CGRP mAb

*Comparison*: placebo

*Outcome*: reduction in days of migraine or headache, reduction in the use of acute attack medication, improvement in function, responder ratio (patients with > 50% reduction in migraine or headache days), serious adverse events (SAEs), mortality (grade of importance: critical)

### Analysis of evidence

We found **15** eligible studies which evaluated whether treatment with CGRP mAbs as compared to placebo is effective and safe [26,27,31-36,41-45,50,**51**]. Among the eligible studies one was on eptinezumab [32], five studies on erenumab [35,36,44,45,50], four studies on fremanezumab [26,27,34,41], and **five** studies on galcanezumab [31,33,42,43]. One phase IIIb study on erenumab was not included in the PICO question 1 because it included only patients with previous drug failure [50].

#### Eptinezumab

Summary of findings for treatment with eptinezumab quarterly injection compared with placebo for prevention of EM is provided in Table 6.

**Table 6:** Summary of findings table for treatment with eptinezumab 1000 mg single intravenous infusion compared with no treatment for prevention of episodic migraine

Outcomes	Anticipated absolute effects^*^(95% CI)		Relative effect(95% CI)	№ of participants (studies)	Certainty of the evidence(GRADE)	Comments
	Risk with placebo	Risk with eptinezumab				
Reduction in migraine days follow up: 3 months	The mean reduction in migraine days was −4.6 days	The mean reduction in migraine days in the intervention group was 1 days fewer (2.1 fewer to 0.2 more)	–	151 (1 RCT)	⨁⨁◯◯ LOW^a^	Treatment with eptinezumab 1000 mg reduces the number of migraine days slightly compared with placebo.
Reduction in use of acute attack medication follow up: 3 months	The mean change in migraines with acute attack medication was + 4.1%	The mean reduction in migraines with acute attack medication was 10.4% days fewer (−20.5% fewer to −0.2% fewer)	–	151 (1 RCT)	⨁⨁◯◯ LOW^a^	Treatment with eptinezumab 1000 mg results in a small possibly unimportant effect in reduction in use of acute attack medication compared with placebo (statistical significance of the differences not tested).
Improvement in function HIT-6 score follow up: 3 months	The mean improvement in function HIT-6 score was −7.7 points	The mean improvement in function HIT-6 score in the intervention group was 2.4 points lower (5.5 lower to 0.7 higher)	–	151 (1 RCT)	⨁⨁◯◯ LOW^a^	Treatment with eptinezumab 1000 mg results in a small possibly unimportant effect in improvement in function assessed by means of the HIT-6 score compared with placebo (statistical significance of the differences not tested).
At least 50% reduction in days of migraine follow up: 3 months	667 per 1000	727 per 1000 (584 to 905)	RR 1.1597 (0.9407 to 1.4076)	151 (1 RCT)	⨁⨁◯◯ LOW^a^	Treatment with eptinezumab 1000 mg results in a small possibly unimportant effect in at least 50% reduction of days of migraine compared with placebo.
Serious adverse events follow up: 6 months	12 per 1000	24 per 1000 (2 to 264)	RR 2.0000 (0.1849 to 21.6193)	163 (1 RCT)	⨁⨁◯◯ LOW^a^	Treatment with eptinezumab 1000 mg results in a small possibly unimportant effect in serious adverse events occurrence compared with placebo.
Mortality follow up: 6 months	0 per 1000	0 per 1000 (0 to 0)	Not estimable	163 (1 RCT)		No deaths occurred during the double-blind treatment phase of the trial.

*CI*: Confidence interval; *RR:* Risk ratio; *RCT*: randomized controlled trial; ^a^Downgraded twice due to inconsistency and imprecision.

GRADE Working Group grades of evidence

High certainty: We are very confident that the true effect lies close to that of the estimate of the effect

Moderate certainty: We are moderately confident in the effect estimate: The true effect is likely to be close to the estimate of the effect, but there is a possibility that it is substantially different

Low certainty: Our confidence in the effect estimate is limited: The true effect may be substantially different from the estimate of the effect

Very low certainty: We have very little confidence in the effect estimate: The true effect is likely to be substantially different from the estimate of effect

A phase II exploratory RCT evaluated the safety and the efficacy of eptinezumab in subjects aged 18–55 years with EM and attack frequency between 5 and 14 days per month [32]. Patients were randomized to a single intravenous injection of eptinezumab 1000 mg or placebo. At weeks 9–12, there was no reduction in migraine days in the eptinezumab compared to the placebo group (mean difference [MD] -1.0; 95% confidence interval [CI] -2.1 to + 0.2). There was a reduction of migraines with acute migraine treatment in the eptinezumab compared to the placebo group (MD -10.4%; 95% CI -20.5 to − 0.2). There was a non-significant improvement in the Headache Impact Test 6 (HIT-6) score in the eptinezumab group compared to the placebo group (MD -2.4; 95% CI -5.5 to + 0.7). The at least 50% responder rate was similar in the eptinezumab and in the placebo group (MD + 10%; 95% CI -4% to + 24%). In the trial, there were 6 SAEs (1 in the placebo group and 5 SAEs in two patients in the erenumab group); the rate of SAEs was 2.4% in the eptinezumab and 1.2% in the placebo group. All the events were deemed to be unrelated to eptinezumab. No deaths were reported.

#### Erenumab

Summary of findings for treatment with erenumab 70 mg monthly injection compared with placebo for prevention of EM is provided in Table 7 and with erenumab 140 mg monthly injection in Table 8.

**Table 7:** Summary of findings table for treatment with erenumab 70 mg monthly subcutaneous injection compared with no treatment for prevention of episodic migraine

Outcomes	Anticipated absolute effects^*^(95% CI)		Relative effect (95% CI)	№ of participants (studies)	Certainty of the evidence (GRADE)	Comments
	Risk with placebo	Risk with erenumab				
Reduction in migraine days follow up: 3–6 months	The mean reduction in migraine days was − 1.9 days	The mean reduction in migraine days in the intervention group was 1.2 days fewer (1.8 fewer to 0.5 fewer)	–	1455 (3 RCTs)	⨁⨁⨁⨁ HIGH	Treatment with erenumab 70 mg results in reduction in migraine days compared with placebo.
Reduction in use of acute attack medication follow up: 3–6 months	The mean reduction in use of acute attack medication was − 0.6 days	The mean reduction in use of acute attack medication in the intervention group was 0.8 days fewer (1.3 fewer to 0.4 fewer)	–	1455 (3 RCTs)	⨁⨁⨁⨁ HIGH	Treatment with erenumab 70 mg results in reduction in use of acute attack medication compared with placebo.
Improvement in functional MPFID everyday-activities follow up: 3–6 months	The mean improvement in functional MPFID everyday-activities was −3.3 points	The mean improvement in functional MPFID everyday-activities in the intervention group was 2.2 points lower (3.3 fewer to 1.2 fewer)	–	628(1 RCT)	⨁⨁⨁◯MEDIUM^a^	Treatment with erenumab 70 mg results in improvement in functional MPFID everyday-activities score compared with placebo.
At least 50% reduction in days of migraine follow up: 3–6 months	283 per 1000	422 per 1000 (366 to 488)	RR 1.4918 (1.2925 to 1.7217)	1441 (3 RCTs)	⨁⨁⨁⨁ HIGH	Treatment with erenumab 70 mg results in at least 50% reduction of days of migraine compared with placebo.
Serious adverse events follow up: 3–6 months	17 per 1000	17 per 1000 (8 to 37)	RR 0.9992 (0.4590 to 2.1752)	1464 (3 RCTs)	⨁⨁⨁⨁ HIGH	Treatment with erenumab 70 mg results in a small possibly unimportant effect in serious adverse events occurrence compared with placebo.
Mortality follow up: 3–6 months	0 per 1000	0 per 1000 (0 to 0)	Not estimable	1464 (3 RCTs)		No deaths occurred during the double-blind treatment phase of the trial.

*The risk in the intervention group (and its 95% confidence interval) is based on the assumed risk in the comparison group and the relative effect of the intervention (and its 95% CI). *CI:* Confidence interval; *RR:* Risk ratio. ^a^Downgraded once due to inconsistency.

GRADE Working Group grades of evidenceHigh certainty: We are very confident that the true effect lies close to that of the estimate of the effect Moderate certainty: We are moderately confident in the effect estimate: The true effect is likely to be close to the estimate of the effect, but there is a possibility that it is substantially different Low certainty: Our confidence in the effect estimate is limited: The true effect may be substantially different from the estimate of the effect Very low certainty: We have very little confidence in the effect estimate: The true effect is likely to be substantially different from the estimate of effect

**Table 8:** Summary of findings table for treatment with erenumab 140 mg monthly subcutaneous injection compared with no treatment for prevention of episodic migraine

Outcomes	Anticipated absolute effects^*^(95% CI)		Relative effect(95% CI)	№ of participants (studies)	Certainty of the evidence(GRADE)	Comments
	Risk with placebo	Risk with erenumab				
Reduction in migraine days follow up: 6 months	The mean reduction in migraine days was −1.8 days	The mean reduction in migraine days in the intervention group was 1.9 days fewer (2.3 fewer to 1.4 fewer)	–	634(1 RCT)	⨁⨁⨁◯MEDIUM^a^	Treatment with erenumab 140 mg results in reduction in migraine days compared with placebo.
Reduction in use of acute attack medicationfollow up: 6 months	The mean reduction in use of acute attack medication was − 0.2 days	The mean reduction in use of acute attack medication in the intervention group was 1.4 days fewer (1.7 fewer to 1.1 fewer)	–	634(1 RCT)	⨁⨁⨁◯MEDIUM^a^	Treatment with erenumab 140 mg results in reduction in number of days of use of acute attack medication compared with placebo.
Improvement in functional MPFID everyday-activities follow up: 6 months	The mean improvement in functional MPFID everyday-activities was − 3.3 points	The mean improvement in functional MPFID everyday-activities in the intervention group was 2.6 points lower (3.6 fewer to 1.5 fewer)	–	634(1 RCT)	⨁⨁⨁◯MEDIUM^a^	Treatment with erenumab 140 mg results in improvement in functional MPFID everyday-activities score compared with placebo.
At least 50% reduction in days of migraine follow up: 6 months	266 per 1000	494 per 1000(353 to 690)	RR 1.8810(1.5191 to 2.3290)	634(1 RCT)	⨁⨁⨁◯MEDIUM^a^	Treatment with erenumab 140 mg results in at least 50% reduction of days of migraine compared with placebo.
Serious adverse events follow up: 6 months	22 per 1000	19 per 1000(6 to 55)	RR 1.0871(0.2913 to 2.5224)	638(1 RCT)	⨁⨁⨁◯MEDIUM^a^	Treatment with erenumab 140 mg results in a small possibly unimportant effect in serious adverse events occurrence compared with placebo.
Mortality follow up: 6 months	0 per 1000	0 per 1000 (0 to 0)	Not estimable	638(1 RCT)		No deaths occurred during the double-blind treatment phase of the trial.

*CI:* Confidence interval; *RR:* Risk ratio; *RCT*: randomized controlled trial; ^a^Downgraded once due to inconsistency.

GRADE Working Group grades of evidenceHigh certainty: We are very confident that the true effect lies close to that of the estimate of the effect Moderate certainty: We are moderately confident in the effect estimate: The true effect is likely to be close to the estimate of the effect, but there is a possibility that it is substantially different Low certainty: Our confidence in the effect estimate is limited: The true effect may be substantially different from the estimate of the effect Very low certainty: We have very little confidence in the effect estimate: The true effect is likely to be substantially different from the estimate of effect

A phase II RCT evaluated the safety and the efficacy of erenumab in subjects aged 18–60 years with EM and attack frequency between 4 and 14 days per month [44]. Patients were randomized to monthly subcutaneous injections of erenumab 70 mg or placebo for 3 months. At 3 month, there was a reduction in monthly migraine days in the erenumab 70 mg compared to placebo group (least squares mean difference [LSMD] –1.1 days; 95% CI –2.1 to − 0.2; *P* = 0.021). There was a reduction in the number of days using acute medication in the erenumab 70 mg compared to the placebo group (LSMD –1.2; 95% CI –2.0 to − 0.3; *P* = 0.006). The at least 50% responder rate was greater in the erenumab 70 mg group compared to the placebo group (46% versus 30%; odds ratio [OR] 2.0; 95% CI 1.2 to 3.4; *P* = 0.011). In the trial, there were 2 SAEs; the rate of SAEs was 0.9% in erenumab 7 mg and 0.9% in erenumab 70 mg group. All the events were deemed to be unrelated to erenumab. No deaths were reported.

A phase III RCT, the STRIVE, evaluated the efficacy of erenumab in subjects aged 18–65 years with EM and attack frequency between 4 and 14 days per month [36]. Patients were randomized to monthly subcutaneous injections of erenumab 70 mg, erenumab 140 mg or placebo for 6 months. At 4–6 months, there was a reduction in monthly migraine days in the erenumab 70 mg (LSMD -1.4; SE -1.9 to − 0.9) and in the erenumab 140 mg (LSMD -1.9; SE -2.3 to − 1.4) groups compared to the placebo group. There was a reduction in the monthly number of days using acute medications in the erenumab 70 mg (LSM -0.9; SE -1.2 to − 0.6) and in the erenumab 140 mg (LSM -1.4; SE -1.7 to − 1.1) groups compared to the placebo group. There was an improvement in the monthly migraine Physical Function Impact Diary (MPFID) everyday-activities score in the erenumab 70 mg (LSMD -2.2; 95% CI -3.3 to − 1.2) and in the erenumab 140 mg (LSMD -2.6; 95% CI-3.6 to − 1.5) groups compared to the placebo group. There was an improvement in the monthly MPFID physical-impairment score in the erenumab 70 mg (LSMD -1.9; 95% CI -3.0 to − 0.8) and in the erenumab 140 mg (LSMD -2.4; 95% CI -3.5 to − 1.4) groups compared to the placebo group. The at least 50% responder rate was greater in the erenumab 70 mg (OR 2.13; 95% CI 1.52 to 2.98) and in the erenumab 140 mg (OR 2.81; 95% CI 2.01 to 3.94) groups compared to the placebo group. In this trial, there were 21 SAEs; the rate of SAEs was 2.5% in the erenumab 70 mg, 1.9% in the erenumab 140 mg, and 2.2% in the placebo group. SAEs were not related to study drug. No deaths were reported.

A phase III RCT, the ARISE, evaluated the efficacy of erenumab in subjects aged 18–65 years with EM and attack frequency between 4 and 14 days per month [35]. Patients were randomized to monthly subcutaneous injections of erenumab 70 mg or placebo for 3 months. At week 12, there was a significant reduction in the monthly migraine days in the erenumab compared to the placebo group (LSMD -1.0; 95% CI -1.6 to − 0.5; *P* < 0.001). There was a significant reduction in the number of days using acute migraine-specific medication (triptan/ergot) in the erenumab compared to the placebo group (LSMD -0.6; SE -1.0 to − 0.2; *P* = 0.002). There was a significant improvement in modified monthly MIDAS total scores in the erenumab compared to the placebo group (LSMD -1.7; SE -3.1 to − 0.3; *P* = 0.021). The at least 50% responder rate per month was greater in the erenumab compared to the placebo group (OR 1.59; 95% CI 1.12 to 2.27; *P* = 0.010). In this trial, there were 8 SAEs; the rate of SAEs was 1.1% in the erenumab 70 mg and 1.7% in the placebo group. No deaths were reported.

#### Fremanezumab

Summary of findings for treatment with fremanezumab 225 mg monthly injection compared with placebo for prevention of EM is provided in Table 9 and with fremanezumab 675 mg quarterly injection in Table 10.

**Table 9:** Summary of findings table for treatment with fremanezumab 225 mg monthly subcutaneous injection compared with no treatment for prevention of episodic migraine

Outcomes	Anticipated absolute effects^*^(95% CI)		Relative effect(95% CI)	№ of participants (studies)	Certainty of the evidence(GRADE)	Comments
	Risk with placebo	Risk with fremanezumab				
Reduction in migraine days follow up: 3 months	The mean reduction in migraine days was − 2.2 days#	The mean reduction in migraine days in the intervention group was 1.7 days fewer (2.6 fewer to 0.8 fewer)	–	776(2 RCT)	⨁⨁⨁⨁HIGH	Treatment with fremanezumab 225 mg results in reduction in migraine days compared with placebo.
Reduction in use of acute attack medication follow up: 3 months	The mean reduction in use of acute attack medication was − 1.6 days#	The mean reduction in use of acute attack medication in the intervention group was 1.5 days fewer (2.3 fewer to 0.6 fewer)	–	776(2 RCT)	⨁⨁⨁⨁HIGH	Treatment with fremanezumab 225 mg results in reduction in use of acute attack medication compared with placebo.
Improvement in functional MIDAS score follow up: 3 months	The mean improvement in functional MIDAS score was − 17.5 points	The mean improvement in functional MIDAS score in the intervention group was 7.6 points lower (14.1 lower to 1.0 lower)	–	776(2 RCT)	⨁⨁⨁⨁HIGH	Treatment with fremanezumab 225 mg results in improvement in functional MIDAS score compared with placebo.
At least 50% reduction in days of migraine follow up: 3 months	269 per 1000	474 per 1000(324 to 693)	RR 1.7594(1.2019 to 2.5754)	776(2 RCT)	⨁⨁⨁⨁HIGH	Treatment with fremanezumab 225 mg results in at least 50% reduction of days of migraine compared with placebo.
Serious adverse events follow up: 3 months	18 per 1000	13 per 1000(4 to 40)	RR 0.7346(0.2352 to 2.2949)	783(2 RCT)	⨁⨁⨁⨁HIGH	Treatment with fremanezumab 225 mg results in small possibly unimportant effect in serious adverse events occurrence compared with placebo.
Mortality follow up: 3 months	0 per 1000	0 per 1000(0 to 0)	not estimable	783(2 RCTs)		No deaths occurred during the double-blind treatment phase of the trials.

#The risk is from a single study; *CI:* Confidence interval; *RR:* Risk ratio; *RCT*: randomized controlled trial

GRADE Working Group grades of evidenceHigh certainty: We are very confident that the true effect lies close to that of the estimate of the effect Moderate certainty: We are moderately confident in the effect estimate: The true effect is likely to be close to the estimate of the effect, but there is a possibility that it is substantially different Low certainty: Our confidence in the effect estimate is limited: The true effect may be substantially different from the estimate of the effect Very low certainty: We have very little confidence in the effect estimate: The true effect is likely to be substantially different from the estimate of effect

**Table 10:** Summary of findings table for treatment with fremanezumab 675 mg quarterly subcutaneous injection compared with no treatment for prevention of episodic migraine

Outcomes	Anticipated absolute effects^*^(95% CI)		Relative effect(95% CI)	№ of participants (studies)	Certainty of the evidence(GRADE)	Comments
	Risk with placebo	Risk with fremanezumab				
Reduction in migraine days follow up: 3 months	The mean reduction in migraine days was − 2.2 days	The mean reduction in migraine days in the intervention group was 1.3 days fewer (1.8 fewer to 0.7 fewer)	–	578(1 RCT)	⨁⨁⨁◯MEDIUM^a^	Treatment with fremanezumab 675 mg results in reduction in migraine days compared with placebo.
Reduction in use of acute attack medication follow up: 3 months	The mean reduction in use of acute attack medication was − 1.6 days	The mean reduction in use of acute attack medication in the intervention group was 1.3 days fewer (1.8 fewer to 0.8 fewer)	–	578(1 RCT)	⨁⨁⨁◯MEDIUM^a^	Treatment with fremanezumab 675 mg results in reduction in use of acute attack medication compared with placebo.
Improvement in functional MIDAS score follow up: 3 months	The mean improvement in functional MIDAS score was − 17.5 points	The mean improvement in functional MIDAS score in the intervention group was 5.4 points lower (8.9 lower to 1.9 lower)	–	578(1 RCT)	⨁⨁⨁◯MEDIUM^a^	Treatment with fremanezumab 675 mg results in improvement in functional MIDAS score compared with placebo.
At least 50% reduction in migraine days follow up: 3 months	279 per 1000	444 per 1000(355 to 557)	RR 1.5912 (1.2700 to 1.9937)	578(1 RCT)	⨁⨁⨁◯MEDIUM^a^	Treatment with fremanezumab 675 mg results in at least 50% reduction of days of migraine compared with placebo.
Serious adverse events follow up: 3 months	24 per 1000	10 per 1000(3 to 39)	RR 0.4330(0.1131 to 1.6582)	584(1 RCT)	⨁⨁⨁◯MEDIUM^a^	Treatment with fremanezumab 675 mg results in small possibly unimportant effect in serious adverse events occurrence compared with placebo.
Mortality follow up: 3 months	0 per 1000	< 1 per 1000(0 to 1)	RR 3.0308(0.1240 to 74.0995)	584(1 RCT)	⨁⨁⨁◯MEDIUM^a^	One death occurred in fremanezumab 675 mg group, and no deaths occurred in the placebo group during the double-blind treatment phase of the trials. Treatment with fremanezumab 675 mg results in small possibly unimportant effect in mortality compared with placebo.

*CI:* Confidence interval; *RR:* Risk ratio; *RCT*: randomized controlled trial; ^a^Downgraded once due to inconsistency.

GRADE Working Group grades of evidenceHigh certainty: We are very confident that the true effect lies close to that of the estimate of the effect Moderate certainty: We are moderately confident in the effect estimate: The true effect is likely to be close to the estimate of the effect, but there is a possibility that it is substantially different Low certainty: Our confidence in the effect estimate is limited: The true effect may be substantially different from the estimate of the effect Very low certainty: We have very little confidence in the effect estimate: The true effect is likely to be substantially different from the estimate of effect

A phase II RCT evaluated the safety and the efficacy of fremanezumab in subjects aged 18–65 years with EM and migraine day frequency between 8 and 14 days per month [27]. Patients were randomized to **three 28-day treatment cycles of subcutaneous 225 mg fremanezumab, 675 mg fremanezumab, or placebo; participants in the 225 mg group received one quarterly injection of 225 mg fremanezumab, while participants in the 675 mg group received 3 monthly injections of 225 mg**. At week 9–12, there was a reduction in migraine days in the fremanezumab 225 mg (LSMD -2.81; 95% CI –4.07 to − 1.55; *p* < 0.0001) and in the fremanezumab 675 mg (LSMD –2.64; 95% CI –3.90 to − 1.38; *P* < 0.0001) groups compared to the placebo group. There was a reduction in the number of days with acute medications in the fremanezumab 225 mg (LSMD -1.76; 95 CI -2.86 to − 0.66; *P* = 0.0018) and in the fremanezumab 675 mg (LSMD -1.70; 95 CI -2.80 to − 0.60; *P* = 0.0026) groups compared to the placebo group. There was an improvement in Migraine Disability Assessment (MIDAS) scores in the fremanezumab 225 mg (LSMD -14.50; 95% -26.79 to − 2.20; *P* = 0.021) and in the fremanezumab 675 mg (LSMD -15.20; 95% -27.62 to − 2.78; *P* = 0.017) groups compared to the placebo group. The at least 50% responder rate was 28% in the placebo group, 53% in the fremanezumab 225 mg group (*P* = 0.0005) and 59% in the fremanezumab 675 mg group (*P* < 0.0001). In this trial, there were 4 SAEs; the rate of SAEs was 2% in the fremanezumab 225 mg and 2% in the fremanezumab 675 mg group. All the events were deemed to be unrelated to fremanezumab. No SAEs were reported in the placebo group. No deaths were reported.

A phase III RCT, the HALO EM, evaluated the safety and the efficacy of fremanezumab in subjects aged 18–70 years with EM and attack frequency between 6 and 14 days per month [34]. Patients were randomized to monthly subcutaneous injections of fremanezumab 225 mg, to quarterly fremanezumab 675 mg, or placebo for 3 months. At 3 months, there was a significant reduction in monthly migraine days in the fremanezumab 225 mg (LSMD –1.5; 95% CI –2.01 to − 0.93; *P* < 0.001) and in the fremanezumab 675 mg (LSMD –1.3; 95% CI –1.79 to − 0.72; *P* < 0.001) groups compared to the placebo group. There was a significant reduction in the monthly number of days using acute medication in the fremanezumab 225 mg (LSMD -1.4; 95% CI –1.84 to − 0.89; *P* < .001) and in the fremanezumab 675 mg (LSMD –1.3; 95% CI –1.76 to − 0.82; *P* < 0.001) groups compared to the placebo group. There was an improvement in mean MIDAS scores in the fremanezumab 225 mg (LSMD –7.0; 95% CI –10.51 to − 3.53; P < 0.001) and in the fremanezumab 675 mg (LSMD –5.4; 95% CI –8.90 to − 1.93; *P* = 0.002) groups compared to the placebo group. The at least 50% responder rate was higher in the fremanezumab 225 mg (difference vs placebo, 19.8%; 95% CI 12.0%–27.6%; *P* < 0.001) and in the fremanezumab 675 mg (difference vs placebo, 16.5%; 95% CI 8.9%–24.1%; *P* < 0.001) groups compared to the placebo group. In this trial there were 13 SAEs; the rate of SAEs was 1.0% in the fremanezumab 225 mg, 1.0% in the fremanezumab 675 mg, and 2.4% in the placebo group. One death occurred in the fremanezumab 675 mg group; the event was considered unrelated to treatment.

#### Galcanezumab

Summary of findings for treatment with galcanezumab 120 mg monthly injection (240 mg loading dose) compared with placebo for prevention of EM is provided in Table 11 and with galcanezumab 240 mg monthly injection in Table 12.

**Table 11:** Summary of findings table for treatment with galcanezumab 240 mg loading dose + 120 mg monthly subcutaneous injection compared with no treatment for prevention of episodic migraine

Outcomes	Anticipated absolute effects^*^(95% CI)		Relative effect(95% CI)	№ of participants (studies)	Certainty of the evidence(GRADE)	Comments
	Risk with placebo	Risk with galcanezumab				
Reduction in migraine days follow up: 6 months	The mean reduction in migraine days was −2.6 days	The mean reduction in migraine days in the intervention group was 2.0 days fewer (2.4 fewer to 1.5 fewer)	–	1330(2 RCT)	⨁⨁⨁⨁HIGH	Treatment with galcanezumab 120 mg results in reduction in migraine days compared with placebo.
Reduction in use of acute attack medication follow up: 6 months	The mean reduction in use of acute attack medication was −2.1 days	The mean reduction in use of acute attack medication in the intervention group was 1.8 days fewer (2.1 fewer to 1.5 fewer)	–	1330(2 RCT)	⨁⨁⨁⨁HIGH	Treatment with galcanezumab 120 mg results in reduction in use of acute attack medication compared with placebo.
Improvement in functional MSQ RFR scorefollow up: 6 months	The mean improvement in functional MSQ RFR score was 22.2 points	The mean improvement in functional MSQ RFR score in the intervention group was 8.3 points higher (6.6 higher to 10.0 higher)	–	1330(2 RCT)	⨁⨁⨁⨁HIGH	Treatment with galcanezumab 120 mg results in improvement in functional MSQ RFR score compared with placebo.
At least 50% reduction in days of migraine follow up: 6 months	372 per 1000	608 per 1000 (543 to 681)	RR 1.6326(1.4578 to 1.8283)	1330(2 RCT)	⨁⨁⨁⨁HIGH	Treatment with galcanezumab 120 mg results in at least 50% reduction of days of migraine compared with placebo.
Serious adverse events follow up: 12–6 months	25 per 1000	58 per 1000 (25 to 135)	RR 2.2738(0.9732 to 5.3128)	1330(2 RCT)	⨁⨁⨁⨁HIGH	Treatment with galcanezumab 120 mg results a small possibly unimportant effect in serious adverse events occurrence compared with placebo
Mortality follow up: 3–6 months	0 per 1000	0 per 1000(0 to 0)	not estimable	1330(2 RCT)		No deaths occurred during the double-blind treatment phase of the trial.

*CI:* Confidence interval; *RR:* Risk ratio; *RCT*: randomized controlled trial;

GRADE Working Group grades of evidenceHigh certainty: We are very confident that the true effect lies close to that of the estimate of the effect Moderate certainty: We are moderately confident in the effect estimate: The true effect is likely to be close to the estimate of the effect, but there is a possibility that it is substantially different Low certainty: Our confidence in the effect estimate is limited: The true effect may be substantially different from the estimate of the effect Very low certainty: We have very little confidence in the effect estimate: The true effect is likely to be substantially different from the estimate of effect.

The mean reduction in migraine days for galcanezumab was changed from 1.9 to 2.0, the mean reduction was changed from "2.3 fewer to 1.3 fewer" to "2.1 fewer to 1.5 fewer" and the mean improvement was modified from 7.7 to 8.3.

The numbers for "at least 50% reduction in days of migraine" was changed from 386 to 372, with modifications throughout the line. The "serious adverse events" are now reported as 25 per 1000 instead of 12 per 1000, with modifications throughout the line.

The number of participants (studies) was changed to 1330 (2 RCT).

The certainty of the evidence was changed from Medium to High

**Table 12:** Summary of findings table for treatment with galcanezumab 240 mg monthly subcutaneous injection compared with no treatment for prevention of episodic migraine

Outcomes	Anticipated absolute effects^*^(95% CI)		Relative effect(95% CI)	№ of participants (studies)	Certainty of the evidence(GRADE)	Comments
	Risk with placebo	Risk with galcanezumab				
Reduction in migraine daysfollow up: 6 months	The mean reduction in migraine days was − 2.6 days	The mean reduction in migraine days in the intervention group was 1.9 days fewer (2.3 fewer to 1.4 fewer)	–	1330(2 RCT)	⨁⨁⨁⨁HIGH	Treatment with galcanezumab 240 mg results in reduction in migraine days compared with placebo.
Reduction in use of acute attack medication follow up: 6 months	The mean reduction in use of acute attack medication was − 2.1 days	The mean reduction in use of acute attack medication in the intervention group was 1.7 days fewer (1.9 fewer to 1.4 fewer)	–	1330(2 RCT)	⨁⨁⨁⨁HIGH	Treatment with galcanezumab 240 mg results in reduction in use of attack medication compared with placebo.
Improvement in functional MSQ RFR score follow up: 6 months	The mean improvement in functional MSQ RFR score was 22.2 points	The mean improvement in functional MSQ RFR score in the intervention group was 7.3 points higher (5.6 higher to 9.1 higher)	–	1330(2 RCT)	⨁⨁⨁⨁HIGH	Treatment with galcanezumab 240 mg results in improvement in functional MSQ RFR score compared with placebo.
At least 50% reduction in days of migrainefollow up: 6 months	372 per 1000	586 per 1000(522 to 658)	RR 1.5738(1.4013 to 1.7675)	1330(2 RCT)	⨁⨁⨁⨁HIGH	Treatment with galcanezumab 240 mg results in at least 50% reduction of days of migraine compared with placebo.
Serious adverse events follow up: 6 months	11 per 1000	16 per 1000(6 to 41)	RR 1.3953(0.5347 to 3.6413)	1330(2 RCT)	⨁⨁⨁⨁HIGH	Treatment with galcanezumab 240 mg results in a small possibly unimportant effect in serious adverse events occurrence compared with placebo.
Mortality follow up: 6 months	0 per 1000	0 per 1000(0 to 0)	not estimable	1330(2 RCT)		No deaths occurred during the double-blind treatment phase of the trial.

*CI:* Confidence interval; *RR:* Risk ratio; *RCT*: randomized controlled trial

GRADE Working Group grades of evidenceHigh certainty: We are very confident that the true effect lies close to that of the estimate of the effect Moderate certainty: We are moderately confident in the effect estimate: The true effect is likely to be close to the estimate of the effect, but there is a possibility that it is substantially different Low certainty: Our confidence in the effect estimate is limited: The true effect may be substantially different from the estimate of the effect Very low certainty: We have very little confidence in the effect estimate: The true effect is likely to be substantially different from the estimate of effect.

The mean reduction in migraine days for galcanezumab was changed from 1.8 to 1.9 the mean reduction was changed from 1.6 to 1.7 and the mean improvement from 7.4 to 7.3.

The numbers for "at least 50% reduction in days of migraine" was changed from 386 to 372, with modifications throughout the line. The "serious adverse events" are now reported as 11 per 1000 instead of 12 per 1000, with modifications throughout the line.

The number of participants (studies) was changed to 1330 (2 RCT).

The certainty of the evidence was changed from Medium to High

A phase II RCT evaluated the safety and the efficacy of galcanezumab in subjects aged 18–65 years with EM and attack frequency between 4 and 14 days per month [33]. Patients were randomized to subcutaneous injections every two weeks of galcanezumab 150 mg or placebo for 3 months. No concomitant preventive medication was allowed. At 9–12 week, there was a reduction in the number of migraine days in the galcanezumab compared to the placebo group (LSMD –1.2; 90% CI –1.9 to − 0.6). There were more at least 50% responder rate in the galcanezumab compared to the placebo group (OR 2.88, 90% CI 1.78–4.69). In this trial, there were 6 SAEs; the rate of SAEs was 1.9% in the galcanezumab and 3.6% in the placebo group. The events were considered unrelated to treatment. No deaths occurred in the study.

A phase IIb RCT evaluated the efficacy of galcanezumab in subjects aged 18–65 years with EM and attack frequency between 4 and 14 days per month [42]. Patients were randomized to subcutaneous injection once a month of galcanezumab 5, 50, 120, or 300 mg or placebo for 3 months. No concomitant preventive medication was allowed. At 9–12 week, there was a greater improvement in migraine days in the galcanezumab 120 mg (− 4.8, 90% Bayesian credible interval [BCI], − 5.4 to − 4.2) compared to the placebo group (− 3.7, 90% BCI, − 4.1 to − 3.2). There was a greater improvement from baseline in the HIT-6 score in the galcanezumab, 120 mg (LSMD − 10.0; 95% CI, − 12.2 to − 7.7; *P* = 0.04) compared to placebo group (LSMD − 7.3; 95% CI − 8.8 to − 5.7). In this trial, there were 4 SAEs; the rate of SAEs was 1.5% in the galcanezumab and 0 in the placebo group. The events were considered unrelated to treatment. No deaths occurred in the study.

A phase III RCT, the EVOLVE-1, evaluated the safety and the efficacy of galcanezumab in subjects aged 18–65 years with EM and attack frequency between 4 and 14 days per month [43]. Patients were randomized to monthly subcutaneous injections of galcanezumab 120 mg (with a loading dose of 240 mg), galcanezumab 240 mg or placebo for 6 months. At 1–6 month, there was a reduction in monthly migraine days averaged over the entire study period in the galcanezumab 120 mg (LSMD -1.9; SE -2.5 to − 1.4; *P* < 0.001) and in the galcanezumab 240 mg (LSMD -1.8; SE -2.3 to − 1.2; *P* < 0.001) group compared to placebo group. There was a reduction in the monthly number of migraine days using acute medication in the galcanezumab 120 mg (LSMD -1.8; SE -2.3 to − 1.3; *P* < 0.001) and in the galcanezumab 240 mg (LSMD -1.6; SE -2.1 to − 1.1; *P* < 0.001) group compared to placebo group. There was an improvement in the MIDAS total score in the galcanezumab 120 mg (LSMD − 21.2; SE 1.7; P < 0.001) and in the galcanezumab 240 mg (LSMD − 20.1; SE 1.7; *P* < .002) groups compared to placebo group (**LSMD -14.9; SE 1.4**). The at least 50% responder rate was greater in galcanezumab 120 mg (OR 2.6; 95% CI 2.0–3.4; P < 0.001) and in galcanezumab 240 mg (OR 2.5; 95% CI 1.9–3.2; *P* < 0.001) groups compared to placebo group. In this trial, there were 12 SAEs in 11 patients; the rate of SAEs was 2.9% in the galcanezumab 120 mg, 0 in the galcanezumab 240 mg, and 1.2% in the placebo group. The events were considered unrelated to treatment. No deaths occurred in the study.

**A further phase III RCT, the EVOLVE-2, evaluated the safety and the efficacy of galcanezumab in subjects aged 18–65 years with EM and attack frequency between 4 and 14 days per month [51]. Patients were randomized to monthly subcutaneous injections of galcanezumab 120 mg (with a loading dose of 240 mg), galcanezumab 240 mg or placebo for 6 months. At 1–6 month, there was a reduction in monthly migraine days averaged over the entire study period in the galcanezumab 120 mg (LSMD -2.02; SE 0.27;** ***P*** **< 0.001) and in the galcanezumab 240 mg (LSMD -1.90; SE 0.27;** ***P*** **< 0.001) group compared to placebo group. There was a reduction in the monthly number of migraine days using acute medication in the galcanezumab 120 mg (LSMD -3.7; SE 0.2;** ***P*** **< 0.001) and in the galcanezumab 240 mg (LSMD -3.6; SE 0.2;** ***P*** **< 0.001) group compared to placebo group (LSMD -1.9; SE 0.2). There was an improvement in the MIDAS total score in the galcanezumab 120 mg (LSMD − 21.2; SE 1.6;** ***P*** **< 0.001) and in the galcanezumab 240 mg (LSMD − 20.2; SE 1.6;** ***P*** **< 0.001) groups compared to placebo group (LSMD -12.0; SE 1.3). The at least 50% responder rate was 36.0% in the placebo group, 59.3% in the galcanezumab 120 mg group (*P*** **< 0.001) and 56.5% in the galcanezumab 240 mg group (*P*** **< 0.001). In this trial, there were 12 SAEs in 11 patients; the rate of SAEs was 2.2% in the galcanezumab 120 mg, 3.1% in the galcanezumab 240 mg, and 1.1% in the placebo group. The events were considered unrelated to treatment. No deaths occurred in the study.**

### Clinical guidance

Available studies indicated that erenumab, fremanezumab, and galcanezumab are effective for prevention in patients with EM. They reduce the number of headache or migraine days, reduce the number of days using acute medications, improve disability. Evidence for erenumab, fremanezumab, and galcanezumab is based on phase II and III RCTs. For eptinezumab benefits are not entirely clear and improvement was significant only in the reduction of medications used for acute attacks; additionally, evidence is based on an exploratory phase II RCT. Eptinezumab is administered via intravenous injection while erenumab, fremanezumab, and galcanezumab are administered via subcutaneous injections. Ease of use represents a potential advantage as CGRP mAbs offer the convenience and adherence benefits of monthly or quarterly dosing allowing avoidance of the daily pill burden. Treatment effect was evident after the first injection and patients continued to improve within the fifth month of treatment [**42**,43, **51**]. The quick onset of action is a potential advantage of CGRP mAbs as compared to conventional treatments. Reduction in migraine days with CGRP mAbs were only modest and ranged from 1 to 2 when compared to placebo. However, the absolute effect of treatment was larger considering also the placebo effect. Perhaps, more clinically significant is the at least 50% responder rate, which was consistently increased with treatment in a clinically meaningful way. A proportion of patients may have a 100% response rate to CGRP mAbs [**37**,39]. The open-label extension of the phase II RCT of erenumab reported low discontinuation rates [24] which is in contrast to current migraine prophylactics that are associated with high discontinuation rates [8,52,53]. Post-hoc analyses of the RCTs indicated that treatment with fremanezumab is associated with improved normal function performance on headache free days [46] and that treatment with galcanezumab is associated with overall functional improvement [23]. At the moment, it cannot be determined whether unique patient populations will have a response to a specific drug.

Data from RCTs indicated that the CGRP mAbs are safe. No relevant SAEs were registered. One death occurred in the phase III RCT on fremanezumab [34] and one death occurred in the open label extension trial on erenumab [24]. Both deaths were considered unrelated to the study drugs. However, it should be noted that further data from the real-life setting are needed to support safety and to provide information on the long-term use.

**PICO question 2:** In patients with CM, is preventive treatment with CGRP mAbs as compared to placebo, effective and safe?

*Population*: patients with CM

*Intervention*: any CGRP mAb

*Comparison*: placebo

*Outcome*: reduction in days of migraine or headache, reduction in the use of acute attack medication, improvement in function, responder ratio (patients with > 50% reduction in migraine or headache days), serious adverse events, mortality (grade of importance: critical)

### Analysis of evidence

We found four eligible studies which evaluated whether treatment with CGRP mAbs as compared to placebo is effective and safe [26,31,41,45]. Among the eligible studies one study was on erenumab [45], two studies on fremanezumab [26,41], and one on galcanezumab [31].

#### Erenumab

Summary of findings for treatment with erenumab 70 mg monthly injection compared with placebo for prevention of CM is provided in Table 13 and with erenumab 140 mg monthly injection in Table 14.

**Table 13:** Summary of findings table for treatment with erenumab 70 mg monthly subcutaneous injection compared with no treatment for prevention of chronic migraine

Outcomes	Anticipated absolute effects^*^(95% CI)		Relative effect(95% CI)	№ of participants (studies)	Certainty of the evidence(GRADE)	Comments
	Risk with placebo	Risk with erenumab				
Reduction of monthly migraine days follow up: 3 months	The mean reduction of monthly migraine days was − 4.2 days	The mean reduction of monthly migraine days in the intervention group was 2.5 days fewer (3.5 lower to 1.4 fewer)	–	469(1 RCT)	⨁⨁⨁◯MEDIUM^a^	Treatment with Erenumab 70 mg reduces monthly migraine days slightly compared to placebo.
Reduction of monthly acute treatment daysfollow up: 3 months	The mean reduction of monthly acute treatment days was −1.6 days	The mean reduction of monthly acute treatment days in the intervention group was 1.9 days fewer (2.6 fewer to 1.1 fewer)	–	469(1 RCT)	⨁⨁⨁◯MEDIUM^a^	Treatment with Erenumab 70 mg reduces monthly acute treatment days slightly compared to placebo.
At least 50% reduction of monthly migraine days follow up: 3 months	235 per 1000	399 per 1000(303 to 525)	RR 1.6985 (1.2908 to 2.2349)	469(1 RCT)	⨁⨁⨁◯MEDIUM^a^	Treatment with Erenumab 70 mg results in at least 50% reduction of monthly migraine days compared to placebo.
Serious adverse events follow up: 3 months	25 per 1000	31 per 1000(11 to 92)	RR 1.2722(0.4340 to 3.7268)	471(1 RCT)	⨁⨁⨁◯MEDIUM^a^	Treatment with Erenumab 70 mg results in a small unimportant increase of serious adverse event occurrence compared to placebo.
Mortalityfollow up: 3 months	0 per 1000	0 per 1000(0 to 0)	not estimable	471(1 RCT)		No deaths were observed with treatment with Erenumab 70 mg or placebo

*CI:* Confidence interval; *RR:* Risk ratio; *RCT*: randomized controlled trial; ^a^Downgraded once due to imprecision: phase II study

GRADE Working Group grades of evidenceHigh certainty: We are very confident that the true effect lies close to that of the estimate of the effect Moderate certainty: We are moderately confident in the effect estimate: The true effect is likely to be close to the estimate of the effect, but there is a possibility that it is substantially different Low certainty: Our confidence in the effect estimate is limited: The true effect may be substantially different from the estimate of the effect Very low certainty: We have very little confidence in the effect estimate: The true effect is likely to be substantially different from the estimate of effect

**Table 14:** Summary of findings table for treatment with erenumab 140 mg monthly subcutaneous injection compared with no treatment for prevention of chronic migraine

Outcomes	Anticipated absolute effects^*^(95% CI)		Relative effect(95% CI)	№ of participants (studies)	Certainty of the evidence(GRADE)	Comments
	Risk with placebo	Risk with erenumab				
Reduction of monthly migraine days follow up: 3 months	The mean reduction of monthly migraine days was − 4.2 days	The mean reduction of monthly migraine days in the intervention group was 2.5 days fewer (3.5 fewer to 1.4 fewer)	–	468(1 RCT)	⨁⨁⨁◯MEDIUM^a^	Treatment with Erenumab 140 mg reduces monthly migraine days slightly compared to placebo.
Reduction of monthly acute treatment days follow up: 3 months	The mean reduction of monthly acute treatment days was −1.6 days	The mean reduction of monthly acute treatment days in the intervention group was 2.6 days fewer (3.3 fewer to 1.8 fewer)	–	468 (1 RCT)	⨁⨁⨁◯MEDIUM^a^	Treatment with Erenumab 140 mg reduces monthly acute treatment days slightly compared to placebo.
At least 50% reduction of monthly migraine days follow up: 3 months	235 per 1000	412 per 1000(314 to 540)	RR 1.7531(1.3359 to 2.3007)	468(1 RCT)	⨁⨁⨁◯MEDIUM^a^	Treatment with Erenumab 140 mg results in at least 50% reduction of monthly migraine days compared to placebo.
Serious adverse events follow up: 3 months	25 per 1000	11 per 1000 (2 to 51)	RR 0.4286(0.0900 to 2.0408)	470(1 RCT)	⨁⨁⨁◯MEDIUM^a^	Treatment with Erenumab 140 mg results in a small unimportant decrease of serious adverse event occurrence compared to placebo.
Mortalityfollow up: 3 months	0 per 1000	0 per 1000(0 to 0)	not estimable	470(1 RCT)		No deaths were observed with treatment with Erenumab 140 mg or placebo

*CI:* Confidence interval; *RR:* Risk ratio; *RCT*: randomized controlled trial; ^a^Downgraded once due to imprecision: phase II study

GRADE Working Group grades of evidenceHigh certainty: We are very confident that the true effect lies close to that of the estimate of the effect Moderate certainty: We are moderately confident in the effect estimate: The true effect is likely to be close to the estimate of the effect, but there is a possibility that it is substantially different Low certainty: Our confidence in the effect estimate is limited: The true effect may be substantially different from the estimate of the effect Very low certainty: We have very little confidence in the effect estimate: The true effect is likely to be substantially different from the estimate of effect

A phase II RCT evaluated the safety and the efficacy of erenumab in subjects aged 18–65 years with CM [45]. Patients were randomized to monthly subcutaneous injection of erenumab 70 mg, erenumab 140 mg or placebo for 3 months. At weeks 9–12, there was a reduction in monthly migraine days in the erenumab 70 mg (LSMD -2.5; SE -3.5 to − 1.4; *P* < 0.0001) and in the erenumab 140 mg (LSMD -2.5; SE -3.5 to − 1.4; P < 0.0001) groups compared to placebo group. There was a reduction in monthly number of days using migraines-specific medication in the erenumab 70 mg (LSMD -1.9; SE -2.6 to − 1.1; P < 0.0001) and in the erenumab 140 mg (LSMD -2.6; SE -3.3 to − 1.8; P < 0.0001) groups compared to the placebo group. The at least 50% responder rate was greater in the erenumab 70 mg (40% versus 23%; OR 2.2; 95% CI 1.5 to 3.3; *P* = 0.0001) and in the erenumab 140 mg (41% versus 23%; OR 2.3; 95% CI 1.6 to 3.5; P < 0.0001) groups compared to the placebo group. In this trial, there were 15 SAEs; the rate of SAEs was 3% in erenumab 70 mg, 1% in erenumab 140 mg, and 2% in the placebo group. No deaths were reported.

#### Fremanezumab

Summary of findings for treatment with fremanezumab 675 mg quarterly injection compared with placebo for prevention of CM is provided in Table 15 and with fremanezumab 225 mg monthly injection (675 loading dose) in Table 16.

**Table 15:** Summary of findings table for treatment with fremanezumab 675 mg quarterly subcutaneous injection compared with no treatment for prevention of chronic migraine

Outcomes	Anticipated absolute effects^*^(95% CI)		Relative effect(95% CI)	№ of participants (studies)	Certainty of the evidence(GRADE)	Comments
	Risk with placebo	Risk with Fremanezumab				
Reduction of monthly headache days follow up: 3 months	The mean reduction of monthly headache days was −2.5 days	The mean reduction of monthly headache days in the intervention group was 1.8 days fewer (2.4 fewer to 1.2 fewer)	–	746(1 RCT)	⨁⨁⨁◯MEDIUM^a^	Treatment with Fremanezumab 675 mg reduces monthly headache days slightly compared to placebo.
Reduction of monthly acute treatment days follow up: 3 months	The mean reduction of monthly acute treatment days was −1.9 days	The mean reduction of monthly acute treatment days in the intervention group was 1.8 days fewer (2.4 fewer to 1.2 fewer)	–	746(1 RCT)	⨁⨁⨁◯MEDIUM^a^	Treatment with Fremanezumab 675 mg reduces monthly acute treatment days slightly compared to placebo.
Improvement in functional HIT-6 score follow up: 3 months	The mean improvement in functional HIT-6 score was −4.5 points	The mean improvement in functional HIT-6 score in the intervention group was 1.9 points fewer (2.9 fewer to 0.9 fewer)	–	746(1 RCT)	⨁⨁⨁◯MEDIUM^a^	Treatment with fremanezumab 675 mg improves functional HIT-6 score slightly compared to placebo.
At least 50% reduction of monthly headache days follow up: 3 months	181 per 1000	376 per 1000(292 to 484)	RR 2.0820(1.6167 to 2.6813)	746(1 RCT)	⨁⨁⨁◯MEDIUM^a^	Treatment with Fremanezumab 675 mg results in at least 50% reduction of monthly headache days compared to placebo.
Serious adverse events follow up: 3 months	16 per 1000	8 per 1000(2 to 32)	RR 0.4987(0.1256 to 1.9792)	751(1 RCT)	⨁⨁⨁◯MEDIUM^a^	Treatment with Fremanezumab 675 mg results in an unimportant reduction of serious adverse event occurrence compared to placebo.
Mortalityfollow up: 3 months	0 per 1000	< 1 per 1000	RR 2.9920 (0.1223 to 73.2174)	751(1 RCT)		No deaths were observed with treatment with Fremanezumab 675 mg or placebo

*CI:* Confidence interval; *RR:* Risk ratio; *RCT*: randomized controlled trial; ^a^Downgraded once due to inconsistency.

GRADE Working Group grades of evidenceHigh certainty: We are very confident that the true effect lies close to that of the estimate of the effect Moderate certainty: We are moderately confident in the effect estimate: The true effect is likely to be close to the estimate of the effect, but there is a possibility that it is substantially different Low certainty: Our confidence in the effect estimate is limited: The true effect may be substantially different from the estimate of the effect Very low certainty: We have very little confidence in the effect estimate: The true effect is likely to be substantially different from the estimate of effect

**Table 16:** Summary of findings table for treatment with fremanezumab 675 mg loading dose + 225 mg monthly subcutaneous injection compared with no treatment for prevention of chronic migraine

Outcomes	Anticipated absolute effects^*^(95% CI)		Relative effect(95% CI)	№ of participants (studies)	Certainty of the evidence(GRADE)	Comments
	Risk with placebo	Risk with fremanezumab				
Reduction of monthly headache days follow up: 3 months	The mean reduction of monthly headache days was −2.5 days#	The mean reduction of monthly headache days in the intervention group was 2.1 days lower (2.6 lower to 1.5 lower)	–	922(2 RCTs)	⨁⨁⨁⨁HIGH	Treatment with Fremanezumab 675/225 mg reduces monthly headache days slightly compared to placebo.
Reduction of monthly acute treatment days follow up: 3 months	The mean reduction of monthly headache days was −4.5 days#	The mean reduction of monthly acute treatment days in the intervention group was 2.4 days lower (3.4 lower to 1.4 lower)	–	922(2 RCTs)	⨁⨁⨁⨁HIGH	Treatment with Fremanezumab 675/225 mg reduces monthly acute treatment days slightly compared to placebo.
Improvement in functional HIT-6 score follow up: 3 months	The mean improvement in functional HIT-6 score was −4.5 points	The mean improvement in functional HIT-6 score in the intervention group was 2.4 days fewer (3.4 fewer to 1.4 fewer)	–	746(1 RCT)	⨁⨁⨁◯MEDIUM^a^	Treatment with fremanezumab 675/225 mg improves functional HIT-6 score slightly compared to placebo.
At least 50% reduction of monthly headache days follow up: 3 months	207 per 1000	431 per 1000 (350 to 530)	RR 2.0857 (1.6948 to 2.5667)	922(2 RCTs)	⨁⨁⨁⨁HIGH	Treatment with Fremanezumab 675/225 mg results in at least 50% reduction of monthly headache days compared to placebo.
Serious adverse events follow up: 1 weeks	15 per 1000	13 per 1000(4 to 38)	RR 0.8516(0.2884 to 2.5150)	928(2 RCTs)	⨁⨁⨁⨁HIGH	Treatment with Fremanezumab 675/225 mg results in an unimportant reduction of serious adverse event occurrence compared to placebo.
Mortalityfollow up: 3 months	0 per 1000	0 per 1000(0 to 0)	not estimable	928(2 RCTs)		No deaths were observed with treatment with Fremanezumab 675/225 mg or placebo

#The risk is from a single study; *CI:* Confidence interval; *RR:* Risk ratio; *RCT*: randomized controlled trial; ^a^Downgraded once due to inconsistency.

GRADE Working Group grades of evidenceHigh certainty: We are very confident that the true effect lies close to that of the estimate of the effect Moderate certainty: We are moderately confident in the effect estimate: The true effect is likely to be close to the estimate of the effect, but there is a possibility that it is substantially different Low certainty: Our confidence in the effect estimate is limited: The true effect may be substantially different from the estimate of the effect Very low certainty: We have very little confidence in the effect estimate: The true effect is likely to be substantially different from the estimate of effect

A phase IIb RCT evaluated the safety, the tolerability, and the efficacy of fremanezumab in subjects aged 18–65 years with CM [26]. Patients were randomized to three 28-day treatment cycles of subcutaneous injections of fremanezumab 225 mg (loading dose 675 mg), fremanezumab 900 mg or placebo. At weeks 9–12, there was a reduction in moderate to severe headache days in the fremanezumab 675/225 mg (LSMD -1.84; 95% CI -3.54 to − 0.14; *P* = 0.0345) and in the fremanezumab 900 mg (LSMD -1.96; 95% CI -3.66 to − 0.26; *P* = 0.0237) groups compared to placebo group. There was a reduction in number of days using acute medication in the fremanezumab 900 mg (LSMD -2.04; 95% CI -3.9 to − 0.2; *P* = 0.027) group compared to placebo group. The at least 50% responder rate considering moderate to severe headaches was greater in the fremanezumab 675/225 mg (OR 2.44; 95% CI 1.3 to 4.5; *P* = 0.004) and in the fremanezumab 900 mg (OR 2.97; 95% CI 1.6 to 5.5; *P* = 0.013) groups compared to placebo group. In this trial, there were 4 SAEs; the rate of SAEs was 1% in the fremanezumab 675/225 mg, 2% in the fremanezumab 900 mg, and 1% in the placebo group. All the events were deemed to be unrelated to fremanezumab. No deaths were reported.

A phase III RCT, the HALO CM, evaluated the efficacy of fremanezumab in subjects aged 18–70 years with CM [41]. Patients who had failed 2 of four clusters of preventive treatments were excluded; migraine preventive drugs were permitted during the study in up to 30% of included patients. Patients were randomized to monthly subcutaneous injections of fremanezumab 225 mg (loading dose of 675 mg), to quarterly fremanezumab 675 mg, or placebo for 3 months. During 12-week period, there was a reduction in the average number of headache days per month in the fremanezumab 675 mg (LSMD -1.8; SE 0.3; *P* < 0.001) and in the fremanezumab 675/225 mg (LSMD -2.1; SE 0.3; *P* < 0.001) groups compared to placebo group. There was a reduction in the monthly number of days using acute medication in the fremanezumab 675 mg (LSMD -1.8; SE 0.3; *P* < 0.001) and in the fremanezumab 675/225 mg (LSMD -2.3; SE 0.3; P < 0.001) groups compared to placebo group. There was an improvement in the HIT-6 score in the fremanezumab 675 mg (LSMD -1.9; SE 0.5; *P* < 0.001) and in the fremanezumab 675/225 mg (LSMD -2.4; SE 0.5; *P* < 0.001) groups compared to placebo group. The at least 50% responder rate was increased in the fremanezumab 675 mg (38%) and in the fremanezumab 675/225 mg (41%) groups compared to placebo group (18%; *P* < 0.001). In this trial, there were 14 SAEs; the rate of SAEs was < 1% in the fremanezumab 675 mg, 1% in the fremanezumab 675/225 mg, and 2% in the placebo group. One SAE lead to discontinuation of the trial. One death occurred in the fremanezumab 675 mg group and was deemed to be unrelated to fremanezumab.

#### Galcanezumab

Summary of findings for treatment with galcanezumab 120 mg monthly injection (240 mg loading dose) compared with placebo for prevention of CM is provided in Table 17 and with galcanezumab 240 mg monthly injection in Table 18.

**Table 17:** Summary of findings table for treatment with galcanezumab 120 mg compared with no treatment for prevention of chronic migraine

Outcomes	Anticipated absolute effects^*^(95% CI)		Relative effect(95% CI)	№ of participants (studies)	Certainty of the evidence(GRADE)	Comments
	Risk with placebo	Risk with galcanezumab				
Reduction of monthly migraine days follow up: 3 months	The mean reduction of monthly headache days was −2.7 days	The mean reduction of monthly headache days in the intervention group was 2.1 days lower (2.9 lower to 1.3 lower)	–	836(1 RCT)	⨁⨁⨁◯MEDIUM^a^	Treatment with Galcanezumab 120 mg reduces monthly migraine days slightly compared to placebo.
Reduction of monthly acute treatment days follow up: 3 months	The mean reduction of monthly headache days was −2.2 days	The mean reduction of monthly acute treatment days in the intervention group was 2.5 days lower (3.3 lower to 1.8 lower)^b^	–	836(1 RCT)	⨁⨁⨁◯MEDIUM^a^	Treatment with Galcanezumab 120 mg reduces monthly acute treatment days slightly compared to placebo.
Improvement in functional MIDAS score follow up: 3 months	The mean improvement in functional MIDAS score was −11.5 points	The mean improvement in functional MIDAS score in the intervention group was 8.7 points lower (16.4 lower to 1.1 lower)	–	836(1 RCT)	⨁⨁⨁◯MEDIUM^a^	Treatment with Galcanezumab 120 mg improves functional MIDAS score compared to placebo.
At least 50% reduction of monthly migraine days follow up: 3 months	149 per 1000	284 per 1000(215 to 375)	RR 1.9112(1.4477 to 2.5232)	836(1 RCT)	⨁⨁⨁◯MEDIUM^a^	Treatment with Galcanezumab 120 mg mg results in at least 50% reduction of monthly headache days compared to placebo.
Serious adverse events follow up: 3 months	7 per 1000	4 per 1000(0 to 34)	RR 0.5288(0.0594 to 4.7092)	836(1 RCT)	⨁⨁⨁◯MEDIUM^a^	Treatment with Galcanezumab 120 mg mg results in a possibly unimportant effect on serious adverse event occurrence compared to placebo.
Mortalityfollow up: 3 months	0 per 1000	0 per 1000(0 to 0)	not estimable	836(1 RCT)		No deaths were observed with treatment with Galcanezumab 120 mg or placebo

*CI:* Confidence interval; *RR:* Risk ratio; *RCT*: randomized controlled trial; ^a^Downgraded once due to inconsistency; ^b^nominall significance, non-significant after multiplicity adjustments

GRADE Working Group grades of evidenceHigh certainty: We are very confident that the true effect lies close to that of the estimate of the effect Moderate certainty: We are moderately confident in the effect estimate: The true effect is likely to be close to the estimate of the effect, but there is a possibility that it is substantially different Low certainty: Our confidence in the effect estimate is limited: The true effect may be substantially different from the estimate of the effect Very low certainty: We have very little confidence in the effect estimate: The true effect is likely to be substantially different from the estimate of effect

**Table 18:** Summary of findings table for treatment with galcanezumab 240 mg compared with no treatment for prevention of chronic migraine

Outcomes	Anticipated absolute effects^*^(95% CI)		Relative effect(95% CI)	№ of participants (studies)	Certainty of the evidence(GRADE)	Comments
	Risk with placebo	Risk with galcanezumab				
Reduction of monthly migraine days follow up: 3 months	The mean reduction of monthly headache days was −2.7 days	The mean reduction of monthly headache days in the intervention group was 1.9 days lower (2.7 lower to 1.1 lower)	–	835(1 RCT)	⨁⨁⨁◯MEDIUM^a^	Treatment with Galcanezumab 240 mg reduces monthly migraine days slightly compared to placebo.
Reduction of monthly acute treatment days follow up: 3 months	The mean reduction of monthly headache days was −2.2 days	The mean reduction of monthly acute treatment days in the intervention group was 2.0 days lower (2.8 lower to 1.3 lower)	–	835(1 RCT)	⨁⨁⨁◯MEDIUM^a^	Treatment with Galcanezumab 240 mg reduces monthly acute treatment days slightly compared to placebo.
Improvement in functional MIDAS score follow up: 3 months	The mean improvement in functional MIDAS score was −11.5 points	The mean improvement in functional MIDAS score in the intervention group was 5.5 points lower (13.1 lower to 2.1 higher)	–	835(1 RCT)	⨁⨁⨁◯MEDIUM^a^	Treatment with Galcanezumab 240 mg does not improve functional MIDAS score significantly compared to placebo.
At least 50% reduction of monthly migraine days follow up: 3 months	149 per 1000	285 per 1000(216 to 377)	RR 1.9181(1.4531 to 2.5321)	835(1 RCT)	⨁⨁⨁◯MEDIUM^a^	Treatment with Galcanezumab 240 mg mg results in at least 50% reduction of monthly headache days compared to placebo.
Serious adverse events follow up: 3 months	7 per 1000	19 per 1000(5 to 70)	RR 2.6534(0.7181 to 9.8049)	835(1 RCT)	⨁⨁⨁◯MEDIUM^a^	Treatment with Galcanezumab 240 mg mg results in a possibly unimportant effect on serious adverse event occurrence compared to placebo.
Mortalityfollow up: 3 months	0 per 1000	0 per 1000(0 to 0)	not estimable	835(1 RCT)		No deaths were observed with treatment with Galcanezumab 240 mg or placebo

*CI:* Confidence interval; *RR:* Risk ratio; *RCT*: randomized controlled trial; ^a^Downgraded once due to inconsistency.

GRADE Working Group grades of evidenceHigh certainty: We are very confident that the true effect lies close to that of the estimate of the effect Moderate certainty: We are moderately confident in the effect estimate: The true effect is likely to be close to the estimate of the effect, but there is a possibility that it is substantially different Low certainty: Our confidence in the effect estimate is limited: The true effect may be substantially different from the estimate of the effect Very low certainty: We have very little confidence in the effect estimate: The true effect is likely to be substantially different from the estimate of effect

A phase III RCT, the REGAIN, evaluated the efficacy of galcanezumab in subjects aged 18–65 years with CM [31]. Patients were randomized to monthly subcutaneous injections of galcanezumab 120 mg (loading dose of 240 mg at baseline), galcanezumab 240 mg, or placebo for 3 months. During the 3-month period, there was a reduction in monthly migraine days in the galcanezumab 120 mg group (LSMD -2.1; 95% CI -2.9 to − 1.3) and with galcanezumab 240 mg (LSMD -1.9; 95% CI -2.7 to − 1.1) compared to placebo groups. There was a reduction in monthly number of days using acute medication use in the galcanezumab 240 mg (LSMD -2.0; 95% CI -2.8 to − 1.3) but not in galcanezumab 120 mg as compared to the placebo group. There was an improvement in the MIDAS score in the galcanezumab 120 mg (LSMD -8.7; 95% CI -16.4 to − 1.1) but not in galcanezumab 240 mg as compared to the placebo group. The at least 50% responder rate was increased in the galcanezumab 120 mg (OR 2.1; 95% CI 1.6–2.8) and in the galcanezumab 240 mg (OR 2.1; 95% CI 1.6–2.8) groups compared to placebo group. In this trial, there were 10 SAEs; the rate of SAE was 0.4% in the galcanezumab 120 mg, 1.8% in the galcanezumab 240 mg, and 0.7% in the placebo group. No deaths were reported.

### Clinical guidance

Available studies indicate that erenumab, fremanezumab, and galcanezumab are effective for prevention in patients with CM. They reduce the number of headache days, reduce the number of days using acute medications, improve disability, and are safe. For erenumab evidence is based on a phase II RCT which however was not a dose finding exploratory study but a RCT to assess safety and efficacy. For fremanezumab evidence is based also on phase II and on a phase III RCT while for galcanezumab it is based on a phase III RCT. Studies included patients with a long history of disease and those who had previously failed two or more preventive medications. The trials did not include patients with more refractory disease such as those who had not had a response to two clusters of preventive medications.

**Clinical question 1:** When should treatment with CGRP mAbs be offered to patients with migraine?

### Analysis of evidence

Characteristics of patients according to migraine duration and previous use of preventive drugs is reported in Tables 1 and 2. In all the trials, included patients had a long migraine history less than 15 years. RCTs included patients who had not tried any previous preventive strategy, patients who had failed or not tolerated other preventatives. Patients considered as drug-resistant where on the other hand excluded.

Referring to the RCTs on EM, the phase II RCT on eptinezumab did not exclude patients according to previous failure of preventive drugs [32]. All the others RCTs in EM excluded patients who failed 2 to 4 categories of preventive drugs. For erenumab, patients who had previous medication failure represented 26% to 40% [35,36,44], for fremanezumab they were 27–33% [27,34] and for galcanezumab 18–19% [43]. A phase IIIb study, the LIBERTY trial, evaluated the efficacy of erenumab 140 mg monthly dose as compared to placebo in patients with EM who had been treated unsuccessfully (in terms of either efficacy or tolerability, or both) with between two and four preventive treatments [50]. At 3 months, significantly more patients in the erenumab group than in the placebo group had a 50% or greater reduction from baseline in the mean number of monthly migraine days. Erenumab was also significantly more efficacious than placebo for all secondary endpoints, including improvements in migraine frequency, medication use, and functional outcomes.

Referring to the RCTs on CM, the phase II RCT on erenumab, excluded patients who had no therapeutic response on an adequate trial of > 3 preventive medications [45]. In the overall study population, 74% of patients had previously received preventive treatments [25]; among them, 48 to 50% of patients had failed ≥2 preventive drugs, 66 to 70% had failed > 1 preventive drug and 30 to 34% had no drug failure; 47 to 52% of patients had previous use of topiramate. OnabotulinumtoxinA injections for migraine prevention were prohibited during the study and for at least 4 months before the start of the baseline phase; 23 to 26% of patients had used onabotulinumtoxinA before study entry. MOH was explicitly allowed. Mean monthly acute migraine-specific drug use days was around 9. Treatment differences for erenumab versus placebo were numerically greater in patients with ≥1 or ≥ 2 failed preventive medications than in patients with no prior treatment failure, particularly for the 140 mg dose [25]. The effect was attributable to a lower placebo effect in patients with prior medications failure than in patients without medications failure.

The phase II and III RCTs on fremanezumab in CM excluded patients who had no therapeutic response of ≥2 preventive medications [26,41]. The phase III study explicitly excluded patients with unremitting headaches (headaches for more than 80% of the time they were awake, and less than 4 days without headache per month); daily headache was acceptable if patients had headaches on less than 80% of the time they were awake on most days [41]. In the study, 28 to 31% of patients had previous use of topiramate. In both trials, onabotulinumtoxinA injections were prohibited during the study and in the 4 [41] or 6 [26] months before study entry. In the phase III study, 13 to 18% of patients had used onabotulinumtoxinA before study entry [41]. Patients with medication overuse headache (MOH) were allowed in the study [26,41]. Days of acute drug use per month ranged from 15 to 16 in one study [26] whereas mean 28-day days of use of any acute headache medications was around 13 in the other study [41].

The phase III RCT on galcanezumab in CM excluded patients who had no therapeutic response of > 3 preventive medications [31]. OnabotulinumtoxinA injections were prohibited during the study. Patients who had failed 2 or 3 previous preventive treatments represented 24–35% of the study population. Patients with MOH were allowed in the study and represented 63–64% of all the study population. In this study patients without headache free days were excluded.

### Clinical guidance

In EM, CGRP mAbs were evaluated both in patients with and without previous drug failure. So far, in most of the available phase II and phase III RCTs, participants with previous failure of as few as 2 preventive medication classes for migraine were excluded. This implies that efficacy can be different for patients with severe, treatment-resistant migraine. Only in the LIBERTY study on erenumab 140 mg monthly patients treated unsuccessfully with between two and four preventive treatments were included. The study confirmed effectiveness of erenumab in this subgroup of patients. However, no results were provided for patients stratified according to previous preventive failure versus non tolerability.

In CM, erenumab, fremanezumab, and galcanezumab were evaluated both in patients with and without previous drug failure. Data on erenumab indicated that the drug is effective even in patients with failure to previous drugs. Patients who had previous use of onabotulinumtoxinA were included in RCTs but no information referring to previous efficacy of onabotulinumtoxinA and response to study treatment is available. Erenumab, fremanezumab, and galcanezumab were not evaluated in patients with CM refractory to current available medical treatments. However, due to the poor quality of life of patients with refractory CM it is reasonable to treat them in daily clinical practice with erenumab, fremanezumab, or galcanezumab. Post-marketing studies are needed to provide information about efficacy of CGRP mAbs in refractory CM.

Costs of the CGRP mAbs are not yet entirely known but they will be higher as compared to costs of the other available drugs. Pharmacogenomics studies should provide analyses to consider the economic impact of those drugs taking into account the overall direct and indirect costs related to untreated migraine or to migraine treated with the available drugs. Differences in reimbursement and regulations among countries will probably be present. Efficacy, safety, good tolerability profile and ease of use may represent advantages of CGRP mAbs drugs which may lead patients to prefer those drugs as first-line options. Rather than only efficacy, CGRP mAbs have advantages referring to side effects and treatment administration. Poor response in patients with migraine may also be attributed to lack of compliance to available medical treatments because of the need of taking multiple doses of the drugs or side effects. CGRP mAbs may represent suitable options for patients who have contraindications to other preventive treatments because of comorbidities or side effects and in patients who have poor compliance to other treatments where strategies to improve compliance have failed. However, due to high costs it will not be possible to offer those drugs to all patients with migraine requiring preventive treatment. At the moment, limiting prescription to patients with prior drug failure may represent a reasonable option until pharmaeconomics studies will provide more data. It is important to point out that patients with multiple drug failures were mostly excluded by RCTs. It is important to note that early treatment of patients with high frequency EM may prevent CM with important impact on individuals and society.

Final recommendations based on experts’ opinions are reported in Table 19.

**Table 19:** Recommendations about the use of anti-calcitonin gene-related peptide monoclonal antibodies in subjects with migraine

Clinical question	Recommendation	Strength of the recommendation
1. When should treatment with anti-CGRP monoclonal antibodies be offered to patients with migraine?	In patients with episodic migraine who have failed at least two of the available medical treatments or who cannot use other preventive treatments because of comorbidities, side effects or poor compliance, we suggest the use of erenumab, fremanezumab, or galcanezumab In patients with chronic migraine who have failed at least two of the available medical treatments or who cannot use other preventive treatments because of comorbidities, side effects or poor compliance, we suggest the use of erenumab, fremanezumab, or galcanezumab	Experts’ opinion
2. How should other preventive treatments be managed when using anti-CGRP monoclonal antibodies in patients with migraine?	In patients with episodic migraine, before starting erenumab, galcanezumab or fremanezumab we suggest to stop oral preventive drugs unless the patient had a previous history of chronic migraine before prevention; in this case, we suggest to add the anti-CGRP monoclonal antibody to the ongoing treatment and to re-assess the need of treatment withdrawal In patients with chronic migraine who are on treatment with any oral drug with inadequate treatment response we suggest to add erenumab, fremanezumab, or galcanezumab and to consider later withdrawal of the oral drug In patients with chronic migraine who are on treatment with onabotulinumtoxinA with inadequate treatment response we suggest to stop onabotulinumtoxinA before initiation of erenumab, fremanezumab, or galcanezumab In patients with chronic migraine who are on treatment with erenumab, fremanezumab, or galcanezumab and who may benefit from additional prevention we suggest to add oral preventive drugs	Experts’ opinion
3. When should treatment with anti-CGRP monoclonal antibodies be stopped in patients with migraine?	In patients with episodic migraine, we suggest to consider to stop treatment with erenumab, fremanezumab, and galcanezumab after 6–12 months of treatments In patients with chronic migraine, we suggest to consider to stop treatment with erenumab, fremanezumab, and galcanezumab after 6–12 months of treatments	Experts’ opinion
4. Should medication overuse be treated before offering treatment anti-CGRP monoclonal antibodies to patients with chronic migraine?	In patients with chronic migraine and medication overuse, we suggest to use erenumab, fremanezumab, and galcanezumab before or after withdrawal of acute medications	Experts’ opinion
5. In which patients anti-CGRP monoclonal antibodies are not to be used?	In patients with migraine, we suggest to avoid anti-CGRP monoclonal antibodies in pregnant or nursing women, in individuals with alcohol or drug abuse, cardio and cerebrovascular diseases, and with severe mental disorders	Experts’ opinion
6. Should binding and/or neutralizing antibodies be monitored?	In patients with migraine on treatment with anti-CGRP monoclonal antibodies, we suggest not to test binding and/or neutralizing antibodies in daily clinical practice; we suggest to further study the possible implications of binding and/or neutralizing antibodies	Experts’ opinion

**Clinical question 2**: How should other preventive treatments be managed when using CGRP mAbs in patients with migraine?

### Analysis of evidence

Summary about concomitant preventive treatments in the RCTs is available in Tables 1 and 2. In the RCT with eptinezumab, preventive drugs were not allowed [32]. In the phase II RCTs on erenumab in EM and CM preventive treatments were not allowed [44,45] whereas in the phase III RCTs preventatives were allowed but a low proportion of patients (2–7%) had concomitant use [35,36]. All RCTs on fremanezumab allowed the inclusion of those patients [26,27,34,41]. Concomitant users of preventive drugs ranged from 20 to 34% for EM [27,34] and from 20 to 43% for CM [26,41]. Notably, a sub-analysis of patients using fremanezumab as an add-on treatment [30] and pooling together data of patients with EM and CM showed that among patients who received fremanezumab as add-on to their preventive treatment there was a significant decrease in the number of migraine days relative to placebo (− 4.1 versus − 2.5), an increase in the number of patients who had improvement by 50% or more of migraine days (40% versus 24%; *P* = 0.0505) and a reduction in the mean number of days using acute medications (33% vs 26%). Patients who were taking oral preventive drugs were not included in the RCTs on galcanezumab in EM [33,42,43,51] but were allowed in the RCT in CM [31].

### Clinical guidance

We have scarce information on how to manage other oral preventive treatments in association with anti-CGRP mAb in patients with migraine. No interaction is supposed by CGRP mAbs and available preventive treatments. Data on erenumab and fremanezumab suggest that the two drugs are beneficial also when added to ongoing oral preventive treatment. Combined use of other prophylactics and CGRP mAbs may be considered in patients with insufficient response to a single type prophylactics. If patients are on preventive drugs that do have some but not sufficient effect, anti-CGRP antibodies can be added because no interaction is expected. When a possible efficacy of anti-CGRP mAb is established in a given patient it should be discussed with the patient whether withdrawal from the oral prophylactic drug should be tried.

In patients with CM, it is reasonable not to stop current ongoing migraine preventive drugs in patients before initiating the use of erenumab, fremanezumab, or galcanezumab in order to avoid possible rebound effects. Withdrawal of other preventive drugs may be done later in patients showing favorable clinical response after starting anti-CGRP mAb. A further point is to clarify, in patients with CM who had favorable response to anti-CGRP mAb but who may continue to experience a significant burden of migraine attacks if adding-on any preventive strategy may further improve attacks frequency, attacks severity, use of preventive drugs and quality of life. At the moment, no such information is available but it is reasonable to allow the use of additional preventive drugs where prevention with anti-CGRP mAb is still considered not optimal.

No information on current use of erenumab, fremanezumab, and galcanezumab with onabotulinumtoxinA is available and this association is not supported at the moment. For those patients who are on botulinum toxin and who show an inadequate response, withdrawal of onabotulinumtoxinA with start of the anti-CGRP mAb may be considered. While in the trials there were time restriction referring to onabotulinumtoxinA withdrawal and start of the anti-CGRP mAb, they represented procedures to avoid confounders and are not reasonable in daily clinical practice. At the moment, we do not know whether it is reasonable to consider combining onabotulinumtoxinA with anti-CGRP mAb in patients who have a suboptimal response to each of those drugs.

Final recommendations based on experts’ opinions are reported in Table 19.

**Clinical question 3**: When should treatment with CGRP mAbs be stopped in patients with migraine?

### Analysis of evidence

For eptinezumab duration of treatment in the available RCT was 3 months [32].

For erenumab in available RCTs duration of treatment ranged from 3 months to 6 months for EM and from 3 months to 3 months for CM [35,36,44,45]. In the open-label extension of the phase II RCT in EM in patients who had completed the 1-year open-label follow-up, persistent benefits were reported for patients who continued treatment up to 1 year [24]. There was a fatal event related to atherosclerosis and a non-fatal myocardial ischemia in patients treated with erenumab 70 mg [24]. The fatal event was considered not related to erenumab while for the other event conclusion was uncertain. A post-hoc analysis of data from the STRIVE and the phase II study in CM, showed evidence of onset of efficacy of erenumab during the first week of treatment [40]. At week 1, 43% of EM patients and 26% of CM patients in the erenumab 140 mg group experienced a ≥ 50% reduction in weekly migraine days (15% increase vs placebo for EM and 10% increase vs placebo for CM). For fremanezumab duration of treatment was of 3 months in all the trials in EM and CM [26,27,34,41]. A post-hoc analysis of patients treated with fremanezumab in the phase II studies indicated that fremanezumab may be associated with sustained efficacy in a substantial percentage of those who show an initial response; sustained response is less obvious in patients with CM than in patients with EM [37]. For galcanezumab duration of treatment in available RCTs in EM was from 3 up to 6 months [31,33,42,43,51]. A pooled analysis of data on galcanezumab in patients with EM (EVOLVE-1 and EVOLVE-2 parallel studies) or CM (REGAIN study) examined the likelihood of response with continued galcanezumab treatment in patients with EM or CM without initial clinical improvement [49]. In patients with EM having “modest” early improvement at month 1, 62% achieved “good” and 20% achieved “better” responses with continued treatment. A percentage of patients with “limited” (43%) or “minimal/no” (34%) early improvement, or “worsening” (20%;) achieved a “good” response after continued treatment. In patients with CM, having “modest” early improvement, 38% achieved “good” and 13% “better” responses with continued treatment. A “good” response was achieved for a percentage of patients with “minimal/no” early improvement (17%). Similar patterns were observed for those without clinical response at month 2, though percentages were lower.

An open label study which evaluated safety and tolerability of galcanezumab 120 and 240 mg in patients with episodic and chronic migraine provided information on treatment at 1-year. In the study authors found high study completion rate (77.8%) supporting the tolerability of the study drug through all 12 months of treatment [47]. In patients who completed the study, treatment compliance was > 95%. Furthermore, the percentage of discontinuations due to adverse events was low (< 5% combined doses), and few SAEs occurred (< 4% combined doses, and none considered related to treatment). A further analysis from this same study indicated that treatment with galcanezumab led to high levels of satisfaction and high levels of preference and less side effects versus previous treatments [48].

### Clinical guidance

As a general rule, treatment can be stopped if migraine is considered too infrequent to justify preventive treatment or if treatment is considered not effective.

Data from the available trials suggest that the effective reduction of monthly headache or migraine days due to treatment with CGRP mAbs may be observed very early, after less than one month from the first dose. Data from RCTs suggest that patients may have additional benefits with continuation of treatment and that some patients who have worsening with treatment or who are considered non-responders may have improvement with continuation of treatment. For those reason it is reasonable not to stop treatment before 3 months even in the absence of a clinical response. Further studies are needed to better assess whether some patients might have even a more delayed response to CGRP mAbs, and to provide information about the durability of the response to treatment with CGRP mAbs. Further data are also needed to clarify whether the response may be sustained even after withdrawal of the CGRP mAbs. For the moment it is reasonable to manage the duration of treatment with CGRP mAbs not differently to other available preventive strategies and to continue it for at least 6–12 in patients who have beneficial effects with those drugs.

Factors contributing to response/nonresponse have yet to be elucidated and clinical judgment should be exercised when deciding whether to discontinue treatment.

Tachyphylaxis of preventive treatments for migraine is a frequent problem in the clinical setting. A post-hoc analysis of patients treated with fremanezumab in the phase II study supported a sustained efficacy, over the 3-month trial period, in a substantial percentage of those who show an initial response [37]. One-year interim analysis of a phase II study of erenumab 70 mg suggest that benefits persist over time [24].

Final recommendations based on experts’ opinions are reported in Table 19.

**Clinical question 4:** Should medication overuse be treated before offering treatment CGRP mAbs to patients with CM?

### Analysis of evidence

All the available RCTs on CM included patients with MOH [26,31,41,45]. No subgroup analysis of efficacy and safety was performed for patients with MOH.

### Clinical guidance

We have no direct data about the impact of MOH on the treatment of CM with CGRP mAbs. However, the available RCTs of erenumab, fremanezumab, and galcanezumab all enrolled consistent proportions of patients with untreated MOH. Therefore, it might be reasonable to offer treatment with CGRP mAbs to patients with MOH. We have, at this moment, no evidence to indicate that the effect of CGRP mAbs is increased if preceded by detoxification and further research is needed on this issue. Some adopt withdrawal strategies before offering preventive medications to patients with CM and MOH and some of the available evidence indicate that detoxification is feasible and effective [54]. However, detoxification is not easy and feasible with all patients and dedicated resources, which are not always available, are needed. We have no data which indicate if the use of CGRP mAbs may favor detoxification in patients with CM and MOH. Of note, the frequent use of butalbital-containing medications was an exclusion criterion from the trials; therefore, current evidence suggests avoiding the overuse of butalbital before starting treatment with CGRP mAbs.

Final recommendations based on experts’ opinions are reported in Table 19.

**Clinical question 5:** In which patients CGRP mAbs are not to be used?

### Analysis of evidence

Criteria varied across trial but pregnant or nursing women, alcohol or drug abuse, cardio and cerebrovascular diseases, and severe mental disorders were the most relevant conditions.

### Clinical guidance

CGRP mAbs are unlikely to produce drug interactions or affect the course of ongoing disease which may be particularly relevant in patients with comorbidities. CGRP is the most potent vasodilator peptide known [55] and has been theoretically considered as dangerous in patients with diseases of the vascular system. In the cardiovascular system, CGRP is present in nerve fibers that innervate blood vessels and the heart and participates in the regulation of blood pressure [56]. For this reason, patients with cardio and cerebrovascular disease were excluded from available clinical trials. In available studies, there is no evidence of increased cardiovascular events or any other serious concerns. However, the duration of available studies is much shorter than the duration in the clinical settings and registries should record any SAEs to see the long-term effects of continuous blockade of the CGRP pathway.

Additionally, there was no effect on treadmill exercise time in patients with angina who received telcagepant, a small-molecule CGRP antagonist [57]. These results supplement those from a placebo-controlled study of erenumab in a high-risk population of patients with stable angina with a median age of 65 years, in which inhibition of the canonical CGRP receptor with erenumab did not adversely affect total exercise time in a treadmill test, among other safety endpoints [58].

Long-term safety studies with CGRP mAbs are needed to further characterize potential cardiovascular effects. More data from migraine patients with comorbid cardiovascular conditions in a real-world setting may help further assess the theoretical cardiovascular risk of blocking the CGRP pathway.

Final recommendations based on experts’ opinions are reported in Table 19.

**Clinical question 6**: Should binding and/or neutralizing antibodies be monitored?

### Analysis of evidence

The occurrence of binding or neutralizing antibodies against the anti-CGRP mAb is reported in Table 20. It is important to note that some of the patients in the RCTs were positive for those antibodies before initiation of the study drug and that there were antibody positive patients even in those taking placebo.

**Table 20:** Binding or neutralizing antibodies directed against anti-CGRP monoclonal antibodies in available randomized clinical trials

Author, Year	Phase, setting	Participants (n)	Follow-up	Binding antibodies	Neutalizing antibodies	Clinical implications
Eptinezumab						
Dodick, 2014 [32]	II, EM	174	3 months	11/81	–	None
Erenumab						
Sun, 2016 [44]	II, EM	483	3 months	8/104 for 70 mg	1/104 for 70 mg	None
Tepper, 2017 [45]	II, CM	667	3 months	11/190 for 70 mg 3/188 for 140 mg	0	None
STRIVE [36]	III, EM	955	6 months	8.0% for 70 mg 3.2% for 140 mg	0.2% for 70 mg 0 for 140 mg	None
ARISE [35]	III, EM	577	3 months	4.3% for 70 mg	0.4% for 70 mg*	None
Fremanezumab						
Bigal, 2015 [27]	IIb, EM	297	3 months	1%§	–	None
Bigal, 2015 [26]	IIb, CM	264	3 months	1%§	–	None
HALO EM [34]	III, EM	875	3 months	1.4% for the monthly dosing 0 for single high dose	–	None
HALO CM [41]	III, CM	1130	3 months	1%	–	None
Galcanezumab						
REGAIN [31]	III, CM	836	3 months	2.7% for 120 mg 2.6% for 240 mg	2.3% for 120 mg 1.5% for 240 mg	None
Dodick, 2014 [33]	II, EM	218	3 months	15.7%#		None
Skljarevski, 2018 [42]	IIb, EM	936	3 months	–	–	None
EVOLVE 1 [43]	III, EM	1671	6 months	3.5% for 120 mg¶ 5.2% for 240 mg¶	0.2%	None
EVOLVE 2 [51]	III, EM	922	6 months	8.6% for 120 mg¶5.1% for 240 mg¶	3.1%	None

* positive at week 4 for but negative at each subsequent visit; §patients were positive at baseline; #including 6.2% of patients who were positive at baseline; ¶only treatment emergent antibodies. The EVOLVE 2 study line under Galcanezumab was replaced with Skljarevski and a new line was added for EVOLVE 2, linked to reference 51

### Clinical guide

Data from individual studies indicate that binding and/or neutralizing antibodies occur infrequently and may have a variable course over time. At the moment, the presence of binding and/or neutralizing antibodies has not been associated with poor response to treatment or adverse events. Consequently, there is no evidence which may support the need of antibodies testing in routine clinical practice. However, this issue should be further studied. In fact, duration of treatment in available studies is limited in time and it cannot be excluded that the rate of occurrence of binding and/or neutralizing antibodies in available clinical studies was too low to establish firm conclusions about their possible implications. Pooled data from available RCTs or data from real life studies may add better evidence and further research should clarify the role of binding and/or neutralizing antibodies in patients with poor clinical response and side effects.

Final recommendations based on experts’ opinions are reported in Table 19.

## References

[1] Sacco S (2019). European headache federation guideline on the use of monoclonal antibodies acting on the calcitonin gene related peptide or its receptor for migraine prevention. J Headache Pain.

